# Proteogenomic analysis of the total and surface-exposed proteomes of *Plasmodium vivax* salivary gland sporozoites

**DOI:** 10.1371/journal.pntd.0005791

**Published:** 2017-07-31

**Authors:** Kristian E. Swearingen, Scott E. Lindner, Erika L. Flannery, Ashley M. Vaughan, Robert D. Morrison, Rapatbhorn Patrapuvich, Cristian Koepfli, Ivo Muller, Aaron Jex, Robert L. Moritz, Stefan H. I. Kappe, Jetsumon Sattabongkot, Sebastian A. Mikolajczak

**Affiliations:** 1 Institute for Systems Biology, Seattle, Washington, United States of America; 2 Center for Infectious Disease Research, Seattle, Washington, United States of America; 3 Department of Biochemistry and Molecular Biology, Center for Malaria Research, Pennsylvania State University, University Park, Pennsylvania, United States of America; 4 Mahidol Vivax Research Unit, Faculty of Tropical Medicine, Mahidol University, Bangkok, Thailand; 5 Population Health and Immunity Division, The Walter and Eliza Hall Institute for Medical Research, Parkville, Victoria, Australia; 6 Malaria: Parasites & Hosts Unit, Institut Pasteur, Paris, France; 7 Faculty of Veterinary and Agricultural Sciences, The University of Melbourne, Parkville, Victoria, Australia; 8 Department of Global Health, University of Washington, Seattle, Washington, United States of America; National Institute of Allergy and Infectious Diseases, UNITED STATES

## Abstract

*Plasmodium falciparum* and *Plasmodium vivax* cause the majority of human malaria cases. Research efforts predominantly focus on *P*. *falciparum* because of the clinical severity of infection and associated mortality rates. However, *P*. *vivax* malaria affects more people in a wider global range. Furthermore, unlike *P*. *falciparum*, *P*. *vivax* can persist in the liver as dormant hypnozoites that can be activated weeks to years after primary infection, causing relapse of symptomatic blood stages. This feature makes *P*. *vivax* unique and difficult to eliminate with the standard tools of vector control and treatment of symptomatic blood stage infection with antimalarial drugs. Infection by *Plasmodium* is initiated by the mosquito-transmitted sporozoite stage, a highly motile invasive cell that targets hepatocytes in the liver. The most advanced malaria vaccine for *P*. *falciparum* (RTS,S, a subunit vaccine containing of a portion of the major sporozoite surface protein) conferred limited protection in Phase III trials, falling short of WHO-established vaccine efficacy goals. However, blocking the sporozoite stage of infection in *P*. *vivax*, before the establishment of the chronic liver infection, might be an effective malaria vaccine strategy to reduce the occurrence of relapsing blood stages. It is also thought that a multivalent vaccine comprising multiple sporozoite surface antigens will provide better protection, but a comprehensive analysis of proteins in *P*. *vivax* sporozoites is not available. To inform sporozoite-based vaccine development, we employed mass spectrometry-based proteomics to identify nearly 2,000 proteins present in *P*. *vivax* salivary gland sporozoites. Analysis of protein post-translational modifications revealed extensive phosphorylation of glideosome proteins as well as regulators of transcription and translation. Additionally, the sporozoite surface proteins CSP and TRAP, which were recently discovered to be glycosylated in *P*. *falciparum* salivary gland sporozoites, were also observed to be similarly modified in *P*. *vivax* sporozoites. Quantitative comparison of the *P*. *vivax* and *P*. *falciparum* salivary gland sporozoite proteomes revealed a high degree of similarity in protein expression levels, including among invasion-related proteins. Nevertheless, orthologs with significantly different expression levels between the two species could be identified, as well as highly abundant, species-specific proteins with no known orthologs. Finally, we employed chemical labeling of live sporozoites to isolate and identify 36 proteins that are putatively surface-exposed on *P*. *vivax* salivary gland sporozoites. In addition to identifying conserved sporozoite surface proteins identified by similar analyses of other *Plasmodium* species, our analysis identified several as-yet uncharacterized proteins, including a putative 6-Cys protein with no known ortholog in *P*. *falciparum*.

## Author summary

Malaria is one of the most important infectious diseases in the world with hundreds of millions of new cases every year. Malaria is caused by parasites of the genus *Plasmodium* which have a complex life cycle, alternating between mosquito and mammalian hosts. Human infections are initiated with a sporozoite inoculum deposited into the skin by parasite-infected mosquitoes as they probe for blood. Sporozoites must locate blood vessels and enter the circulation to reach the liver where they invade and grow in hepatocytes. In the case of *Plasmodium vivax*, one of the two *Plasmodium* species responsible for the majority of the disease burden in the world, the parasite has the ability to persist for months in the liver after the initial infection and its activation causes the recurring appearance of the parasite in the blood. Though all clinical symptoms are attributable to the blood stages, it is only by attacking the transmission stages before the formation of hypnozoites (the persisting parasites in the liver) that an impact on the burden of vivax malaria can be achieved. We used state-of-the-art mass spectrometry-based proteomics tools to identify the total protein make-up of *P*. *vivax* sporozoites. By analyzing which proteins are exposed to the parasite surface and determining the degree of protein’s post-translational modifications, our investigation will aid the understanding of the novel biology of sporozoites and importantly, advise the development of potential vaccine candidates targeting this parasite stage.

## Introduction

Malaria is a major global infectious disease, responsible for nearly 429,000 deaths and 212 million new cases annually (World Malaria Report 2016, WHO). This disease, found in much of the tropical and subtropical regions of the world, is caused by parasites of the genus *Plasmodium*, transmitted to humans by the bite of infected anopheline mosquitoes. Parasites (sporozoites) that have infected the mosquito salivary gland are transmitted to the human host as the mosquito injects saliva while taking a blood meal. These sporozoites find their way to the liver where they invade hepatocytes and reproduce asexually. The mature liver stages rupture and the release of exoerythrocytic merozoites that are ready to invade erythrocytes causes the clinical symptoms of malaria.

The majority of human malaria cases are caused by *P*. *falciparum* and *P*. *vivax*. A large proportion of malaria research efforts focus on *P*. *falciparum* infections, motivated by the severity of clinical symptoms and the high mortality rate that is especially evident among children in sub-Saharan Africa. In contrast, *P*. *vivax* malaria affects more people in a wider global range [1, 2], but infections with *P*. *vivax* often do not cause disease that matches the severity observed for *P*. *falciparum* infections. *P*. *vivax*-infected individuals of all age groups may still endure repeated, debilitating febrile attacks, severe anemia, and respiratory distress that are more frequently fatal than previously appreciated [3]. Additionally, with only one exposure to infectious mosquito bite, *P*. *vivax* can initiate not only one symptomatic infection but a series of reoccurring onsets of malaria episodes that, if not treated, can last for months. These recurring infections are due to a distinctive property of *P*. *vivax* liver infection: formation of hypnozoites, a portion of *P*. *vivax* liver-stage parasites that becomes dormant and can reactivate weeks to months or even years later [4].

The malaria elimination strategies of vector control and treating symptomatic blood-stage infection with anti-malarial drugs are not as effective against *P*. *vivax* as against *P*. *falciparum* because *P*. *vivax* can persist in the liver as dormant hypnozoites, and because *P*. *vivax* gametocytes develop earlier and can be transmitted before onset of clinical symptoms [5–7]. Currently, the only approved treatment for *P*. *vivax* is primaquine. Primaquine, however, comes with major complications: its short half-life translates to long dosage regimens, its toxicity for patients with glucose-6-phosphate-dehydrogenase deficiency requires pre-screening of recipients [8], and limited effectiveness in patients with certain cytochrome P450 2D6 polymorphisms will require consideration [9].

An alternative route to reducing the burden of vivax malaria would be the development of an effective vaccine against *P*. *vivax*. Targeting *P*. *vivax* pre-erythrocytic stages (the sporozoite stage and the liver stage) for vaccine development not only has the advantage that these initial stages of infection involve only a small number of parasites and are completely asymptomatic, but also that such a vaccine could prevent relapsing infections. In fact, one of the most effective experimental vaccination strategies against *P*. *falciparum* infection is the use of live attenuated sporozoites (damaged by irradiation) that are effective in inducing complete immune protection by their ability to mount humoral and cellular immune responses against the sporozoite and the liver stage of the parasite [10]. This method of vaccination was recently tested in *P*. *vivax* showing encouraging protective efficacy [11]. Nevertheless, the major obstacle for a successful pre-erythrocytic vaccine lies in the required threshold for vaccine efficacy: to protect against infection, the pre-erythrocytic vaccine must be completely effective. Full development of a single liver-stage parasite and exoerythrocytic merozoite release results in full-blown blood stage infection and all the clinical consequences of the disease. This requirement–inducing sterile immunity by targeting the liver stages–may be especially difficult to achieve for vaccines targeting the liver stages of *P*. *vivax* due to the ability of the parasite to form hypnozoites. It is presently unknown if vaccination regimens that target developing liver stages would also be able to target hepatocytes harboring hypnozoites.

Thus, an effective subunit vaccination strategy that targets the parasite before it enters the hepatocyte could be the most plausible solution for preventing a *P*. *vivax* hepatocyte infection, development of liver-stage parasites, and hypnozoite formation. It has been shown that antibody responses against the circumsporozoite protein (CSP), a major surface protein on the *Plasmodium* sporozoite, can lead to sterile protection against infection, but in most cases these responses offer only partial protection in *P*. *falciparum* [12]. A recent clinical study in which a *P*. *vivax* CSP-based subunit vaccine was used showed no sterile protection, but a significant delay in the onset of parasitemia was observed [13]. As opposed to *P*. *falciparum* infection where partial protection offers only limited benefits, partial protection that could be observed after immunizations against *P*. *vivax* has the potential to considerably affect the hypnozoite burden in the liver by limiting the number of sporozoites reaching the hepatocyte and developing into hypnozoites, thereby directly decreasing the chances of relapse malaria [14]. A vaccine targeting *P*. *vivax* sporozoites is therefore highly desirable.

The identification of non-CSP antigens that can be included into a multi-antigen subunit vaccine has recently gained momentum for *P*. *falciparum*, but such an effort has not yet been initiated for *P*. *vivax*. After mosquito transmission, sporozoites embark on a complex route of infection in the human host and three biological activities of the sporozoite are essential for their success, namely, gliding motility, cell traversal, and cell invasion. All of these activities require engagement of sporozoite surface and secreted proteins with the host environment and thus might be blocked by antibodies. Therefore, the discovery of new *P*. *vivax* sporozoite surface antigens, together with CSP-based antigens, may allow the development of a better antibody-based, anti-infection vaccine [15].

Mass spectrometry (MS)-based proteomics has previously been employed to catalogue the protein complement of *P*. *falciparum*, *P*. *yoelii* and *P*. *berghei* salivary gland sporozoites with the goal of identifying new targets for therapeutics and new antigens for subunit-based vaccines [16–20]. The most comprehensive proteomic analyses to-date of *P*. *falciparum* salivary gland sporozoites detected over 2000 of the approximately 5000 gene products predicted from the *Plasmodium falciparum* genome [19] and identified 42 putatively surface-exposed sporozoite proteins [20] by a chemical labeling strategy. Here, by applying similar proteomics techniques to the analysis of the proteins present in *P*. *vivax* salivary gland sporozoites of field isolates, a combined total of 1970 *P*. *vivax* proteins were identified, of which 36 have been categorized as putative sporozoite surface proteins. Post-translational modification of sporozoite proteins by glycosylation and phosphorylation have also been evaluated to further aid the development of subunit vaccines.

## Materials and methods

### Ethics statement

The human blood collection protocol was approved by the Ethical Committee of the Faculty of Tropical Medicine, Mahidol University. All adult subjects participating in this study provided written informed consent. No child participants were included in this study.

### Production of *Plasmodium vivax* sporozoite-infected mosquitoes

*Anopheles dirus* mosquitoes (from the Mahidol University colony maintained at the Faculty of Tropical Medicine laboratories) were infected with blood collected from patients who were confirmed positive for only *P*. *vivax* malaria via microscopy at local health centers in close proximity to the Kanchanaburi Campus, Mahidol University. In brief, 150 μL of red blood cell pellet from blood samples was suspended in pooled normal AB serum to a packed cell volume of 50%. The suspension was fed for 30 min to 100 female mosquitoes (5-7 days old) via an artificial membrane attached to a water-jacketed glass feeder maintained at 37°C. Unfed mosquitoes were removed and fed mosquitoes were maintained on a 10% w/v sucrose solution and incubated at 26°C and 80% humidity for at least 14 days. Salivary gland dissections were performed at days 14-19. CSP haplotype (VK210 or VK247) was determined by PCR.

### Sporozoite isolation, purification and surface labeling

Salivary glands from *P*. *vivax*-infected mosquitoes were harvested by microdissection and homogenized by grinding. Sporozoite preparations were purified from mosquito debris on an Accudenz discontinuous gradient as previously described [21]. Total sporozoite numbers were counted on a hemocytometer. For the total proteome analyses, 3.5 × 10^6^ VK210 and 4.5 × 10^6^ VK247 sporozoites were pelleted for 3 min at 16,000 × *g*, re-suspended in 1 × PBS pH 7.4, pelleted, and stored at -80°C. Prior to protein separation by SDS-PAGE, the pellet was re-suspended in an equal volume of 2 × sample buffer and heated for 5 min at 70°C. For the surface proteome samples, 2 × 10^6^ VK210 and 1.8 × 10^7^ VK247 sporozoites were pelleted for 3 min at 4,000 × *g* at 4°C and re-suspended with ice-cold 1 × PBS pH 8.0. The VK247 sample was evenly split into two tubes and all three samples were pelleted again. One of the VK247 samples was set aside as an unlabeled control. The remaining two samples were re-suspended in 40 μL ice-cold 1 × PBS pH 8.0 per 10^6^ sporozoites. A 10 mM stock solution of EZ-Link Sulfo-NHS-SS-Biotin (Thermo Fisher Scientific, part number 21331) was added to a final concentration of 2 mM and the samples were incubated for 1 h at 4°C. The sporozoites were pelleted and re-suspended in 500 μL ice-cold 1 × Tris-buffered saline (TBS) pH 8.0 and incubated for 5 min on ice to quench excess biotin label. The sporozoites were then pelleted for 2 min at 16,000 × *g* and washed a second time in 1 × TBS, each time removing as much supernatant as possible without disturbing the pellet. The samples were stored at -80°C until lysis. The sporozoites were lysed by re-suspending the pellet in 100 μL lysis buffer (1% w/v SDS, 4 M urea, 50 mM Tris-HCl pH 8.0, 150 mM NaCl, 1 × protease inhibitor (Roche cOmplete)) and incubating for 30 min at 4°C with end-over-end rotation. The samples were diluted to 1 mL in 1 × PBS pH 7.4, added to 1 mg of magnetic streptavidin beads (Dynabeads MyONe Streptavidin T1) which had been washed three times in 1 × PBS, and incubated for 1 h at 4°C with end-over-end rotation. The beads were washed sequentially with the following: 1) 2% w/v SDS; 2) 0.1% w/v SDS, 6 M urea, 1 M NaCl, 50 mM Tris-HCl pH 8.0; 3) 0.1% w/v SDS, 4 M urea, 200 mM NaCl, 1 mM EDTA, 50 mM Tris-HCl pH 8.0; 4) 0.1% w/v SDS, 50 mM NaCl, 50 mM Tris-HCl pH 8.0. Bound proteins were eluted by adding 40 μL 2 × sample buffer (4% w/v SDS, 125 mM Tris-HCl pH 6.8, 20% v/v glycerol, 0.02% w/v bromophenol blue) to which dithiothreitol (DTT) was added to a final concentration of 50 mM and heating the tube for 7 min at 70°C. The eluted sample was transferred to a new tube and stored at -80°C until separation by SDS-PAGE.

### SDS-PAGE fractionation

SDS-PAGE pre-fractionation and in-gel tryptic digestion were performed essentially as described in [19]. Extended methods are provided in S1 File. Briefly, samples were electrophoresed through a 4-20% w/v SDS-polyacrylamide gel (Pierce Precise Tris-HEPES). Gels were stained with Imperial Stain (Thermo Fisher Scientific), de-stained in Milli-Q Water (Millipore), and cut into fractions (S1 Table). Gel pieces were then de-stained with 50 mM ammonium bicarbonate (ABC) in 50% acetonitrile (ACN) and dehydrated with ACN. Disulfide bonds were reduced with 10 mM DTT and cysteines were alkylated with 50 mM iodoacetamide in 100 mM ABC. Gel pieces were washed with ABC in 50% ACN, dehydrated with ACN, and rehydrated with 6.25 ng/μL sequencing grade trypsin (Promega). The supernatant was recovered and peptides were extracted by incubating the gel pieces with 2% v/v ACN/1% v/v formic acid, then ACN. The extractions were combined with the digest supernatant, evaporated to dryness in a rotary vacuum, and reconstituted in liquid chromatography (LC) loading buffer consisting of 2% v/v ACN/0.2% v/v trifluoroacetic acid (TFA).

### Liquid chromatography-mass spectrometry

LC and MS parameters were essentially as described previously [19, 20]. Extended method details are provided in S1 Table. Briefly, LC was performed using an Agilent 1100 nano pump with electronically controlled split flow or a Proxeon Easy nLC. Peptides were separated on a column with an integrated fritted tip (360 μm outer diameter (O.D.), 75 μm inner diameter (I.D.), 15 μm I.D. tip; New Objective) packed in-house with a 20 cm bed of C18 (Dr. Maisch ReproSil-Pur C18-AQ, 120 Å, 3 μm). Prior to each run, sample was loaded onto a trap column consisting of a fritted capillary (360 μm O.D., 150 μm I.D.) packed with a 1 cm bed of the same stationary phase and washed with loading buffer or buffer A (0.1% v/v formic in water). The trap was then placed in-line with the separation column for the separation gradient. The LC mobile phases consisted of buffer A and buffer B (0.1% v/v formic acid in ACN). The separation gradient was 5% B to 35% B over 60 min for the surface-labeled samples and 5% B to 25% B over 120 or 180 min for the whole proteome samples. Tandem MS (MS/MS) was performed with an LTQ Velos Pro-Orbitrap Elite (Thermo Fisher Scientific). Data-dependent acquisition was employed to select the top precursors for collision-induced dissociation (CID) and analysis in the ion trap. Dynamic exclusion and precursor charge state selection were employed. Two nanoLC-MS technical replicates were performed for each fraction, with roughly half the available sample injected for each replicate.

### Peak list generation

The MS data generated for this manuscript, along with the search parameters, analysis parameters and protein databases can be downloaded from PeptideAtlas (www.peptideatlas.org) using the identifiers PASS00976 (whole proteome) and PASS00977 (surface-labeled). Mass spectrometer output files were converted to mzML format using msConvert version 2.2.0 (whole proteome data) or 3.0.5533 (surface-labeled data) [22] and searched with Comet version 2015.02 rev.0 [23]. The protein sequence database is described in the following section. The precursor mass tolerance was ±10 ppm, and fragment ions bins were set to a tolerance of 1.0005 *m/z* and a monoisotopic mass offset of 0.4 *m/z*. Semi-tryptic peptides and up to 2 missed cleavages were allowed. The search parameters included a static modification of +57.021464 Da at Cys for formation of S-carboxamidomethyl-Cys by iodoacetamide and potential modifications of +15.994915 Da at Met for oxidation, -17.026549 Da at peptide N-terminal Gln for deamidation from formation of pyroGlu, -18.010565 Da at peptide N-terminal Glu for loss of water from formation of pyroGlu, -17.026549 Da at peptide N-terminal Cys for deamidation from formation of cyclized N-terminal S-carboxamidomethyl-Cys, and +42.010565 for acetylation at the N-terminus of the protein, either at N-terminal Met or the N-terminal residue after cleavage of N-terminal Met. Additionally, the search parameters for sporozoite surface samples included a potential modification of +145.019749 Da at Lys for addition of the biotin label, the disulfide bond of which had been cleaved and alkylated. The MS/MS data were analyzed using the Trans-Proteomic Pipeline (TPP) [24] version 5.0.0 Typhoon. Peptide spectrum matches (PSMs) were assigned scores in PeptideProphet [25], peptide-level scores were assigned in iProphet [26], and Protein identifications were inferred with ProteinProphet [27]. Additional TPP parameters are available in S1 File. In the case that multiple proteins were inferred at equal confidence by a set of peptides, the inference was counted as a single identification and all relevant protein IDs were listed. Only proteins with ProteinProphet probabilities corresponding to a false discovery rate (FDR) less than 1.0% (as determined from the ProteinProphet mixture models) were reported. For comparison with *P*. *falciparum* salivary gland sporozoites, a publically available data set [19] (available from PeptideAtlas using the identifier PASS00095) was re-analyzed with the same software and parameters described above. The spectra were searched against a database comprising *P*. *falciparum* 3D7 [28] (PlasmoDB v.30, www.plasmodb.org [29]), *Anopheles stephensi* Indian AsteI2.3 [30] (VectorBase, www.vectorbase.org [31]), and a modified version of the common Repository of Adventitious Proteins (v.2012.01.01, The Global Proteome Machine, www.thegpm.org/cRAP) with the Sigma Universal Standard Proteins removed and the LC calibration standard peptide [Glu-1] fibrinopeptide B appended. Decoy proteins with the residues between tryptic residues randomly shuffled were generated using a tool included in the TPP and interleaved among the real entries. *P*. *falciparum* protein annotations were updated from PlasmoDB v.32.

### Compiling a reference proteome

A protein database containing sequence polymorphisms of *P*. *vivax* proteins occurring in Thailand was created by aligning DNA-seq and RNA-seq reads from field isolates to the *P*. *vivax* Sal-1 genome [32] (PlasmoDB v.26). DNA-seq reads from 19 Thai field isolates were obtained from www.plasmodb.org and aligned using Burrows Wheeler Aligner (v.0.7.12) and SNVs were called using the Genome Analysis Toolkit (v.3.6). RNA-seq reads for 13 isolates [33] (obtained from https://www.ncbi.nlm.nih.gov/bioproject/, accession number PRJNA376620) were aligned using STAR (v.2.5) and SNVs were called using the Genome Analysis Toolkit (v.3.6). All proteins with sequences different from the Sal-1 reference proteome were compiled (S2 File) and added to a database comprising *P*. *vivax* Sal-1 [32] (PlasmoDB v.31), *P*. *vivax* P01 [34] (PlasmoDB v.31), *Anopheles stephensi* Indian AsteI2.3 [30] (VectorBase), and the modified version of the cRAP proteins described above. Decoy proteins were generated as above. Mass spectra from the two whole-proteome samples and the two surface-labeled samples were searched against the database with Comet as described above and the resulting PSMs were analyzed with PeptideProphet and iProphet as described above except that the NSP model was enabled in iProphet. All *P*. *vivax* peptides identified with iProphet probabilities corresponding to a model-estimated FDR less than 1.0% were aligned against the *P*. *vivax* P01 reference proteome. A new *P*. *vivax* P01 reference proteome was assembled incorporating polymorphism-bearing peptides identified by the above-described search. If a detected peptide was associated with a given *P*. *vivax* protein in at least one of the field isolates but did not align with the *P*. *vivax* P01 reference sequence due to sequence polymorphisms, the variant peptide sequence was appended to the end of the reference protein sequence entry in the fasta database. This modified *P*. *vivax* P01 reference proteome was added to the *An*. *stephensi* and cRAP databases described above. Additionally, the entry for CSP (PVP01_0835600), which contains the tandem repeat region specific to the VK210 haplotype, was appended with the sequence of the VK247 tandem repeat region [35]. Decoys were generated as above. This database was used for all subsequent analysis of the MS data. *P*. *vivax* protein annotations were updated from PlasmoDB v.32.

### Protein quantification

Relative protein abundance within and between samples was estimated using a label-free proteomics method based on spectral counting. Extended method details are provided in S1 File. The spectral counts for a protein were taken as the total number of high-quality PSMs (identified at a PeptideProphet probability corresponding to an FDR less than 1.0%) that identified the protein. PSMs from degenerate peptides (peptides whose sequences were found in multiple proteins in the database) were split among proteins containing that peptide in a weighted fashion [36, 37]. Relative protein abundance within samples was ranked using the normalized spectral abundance factor. The spectral abundance factor (SAF) for a given protein was calculated as the quotient of the total PSMs identifying that protein and the protein's length. The SAF for each *Plasmodium* protein was normalized to the sum of all *Plasmodium* SAF values obtained from the same sample, and this normalized SAF (NSAF) was natural log-transformed to ln(NSAF) [38, 39]. The population of ln(NSAF) values for each sample assumed a normal distribution, as did the population of log-transformed protein abundance fold-change ratios between samples, calculated as ln(NSAF)_A_-ln(NSAF)_B_ where A and B are two different samples in which the same protein was observed. Each of these distributions was fit with a Gaussian curve in Microsoft Excel using minimum residual sum of squares and goodness-of-fit was evaluated with the R^2^ coefficient of determination (S1 and S2 Figs). To assess the relative abundance of proteins between the two samples, PSM counts for all proteins were first increased by 1 in order to assign non-zero values to proteins detected in one sample but not the other [40]. These adjusted spectral counts were then normalized so that the sum of all PSMs was the same in both samples. The abundance ratio for a given protein between a two samples was then calculated as

$$R_{A:B}=\frac{c_{A}}{c_{B}}$$

Where *R*_*A*:*B*_ is the protein abundance ratio of a protein between sample A and sample B and *c*_*A*_ and *c*_*B*_ are the adjusted and normalized spectral counts for the protein in sample A and sample B, respectively. In order to assess the error in spuriously large protein ratios obtained from proteins with low spectral counts, the G-test of significance was applied to the adjusted and normalized spectral counts for each protein pair as

$$G=2\left[c_{A}ln\left(\frac{c_{A}}{\left(\frac{c_{A}+c_{B}}{2}\right)}\right)+c_{B}ln\left(\frac{c_{B}}{\left(\frac{c_{A}+c_{B}}{2}\right)}\right)\right]$$

and a *p*-value was assigned by calculating the probability that a *χ*^2^ distribution with one degree of freedom was more extreme than the G statistic [40]. The distribution of the *log*_2_(*R*_*A*:*B*_) values of all proteins detected in both samples was fit with a Gaussian curve as above. Protein abundance ratios were corrected for systematic bias by subtracting the mean of this distribution (which was near 0 in all cases) from each log-transformed protein ratio. In order to assess the probability that a protein ratio was more extreme than the normal distribution of protein ratios, a *p*-value was calculated for each ratio using the complementary error function as

$$p=ERFC\left|\frac{log_{2}(R_{A:B})-\mu }{\sigma \sqrt{2}}\right|$$

where *μ* is the mean and *σ* is the standard deviation of the fit Gaussian. The FDR arising from multiple hypothesis testing was assessed by the Benjamini-Hochberg method for both tests independently, and protein ratios with an FDR less than 5.0% by both the G-test and ERFC were considered significant.

### Identifying phosphorylated proteins

Phosphorylated peptides were identified by searching the MS data with the same parameters listed above with the additional potential modification mass of +79.966331 Da at Ser, Thr, and Tyr. The PSMs generated from these searches were analyzed separately by PeptideProphet as above, except that the DECOYPROBS option was used so that decoy peptides were assigned probabilities and included in the output. Decoy peptides were used to calculate an FDR among the subset of PSMs exhibiting phosphorylation. Due to the infrequent occurrence of phosphopeptides in these un-enriched samples, the decoy-estimated FDR for phosphopeptide PSMs was as high as 24% in the VK210 sample and 19% in the VK247 sample at the probabilities corresponding to a 1.0% decoy-estimated FDR for the entire population of PSMs. The more stringent cut-off to achieve 1.0% FDR among phosphopeptide PSMs was used to identify high-confidence phosphopeptides. Phosphopeptide PSMs within each sample were only counted if the phosphopeptide was identified by at least one PSM at the high-stringency cut-off and by at least two PSMs in the population-level cut-off. The number of PSMs identifying a phosphopeptide and the number of PSMs identifying the same peptide in un-phosphorylated form were used to estimate the percentage of that peptide that was phosphorylated in the sample. Localization of phosphate groups within phosphopeptides was confirmed and/or corrected using a development version of PTMProphet (source code available at https://sourceforge.net/p/sashimi, SVN revision number 7584. See S1 File for complete parameters).

### Prioritizing proteins identified from surface labeling

Experimental and theoretical evidence was used to identify high-confidence putatively surface-exposed proteins from among those *P*. *vivax* proteins identified by surface labeling live sporozoites with the biotin tag. Proteins were taken for further consideration if they were identified by at least two peptides and three PSMs in at least one of the two labeled samples. Proteins were considered high-quality candidates if they possessed predicted characteristics of a surface protein, i.e., transmembrane (TM) domain(s), a signal peptide, or a glycophosphatidylinositol (GPI) anchor, or if they exhibited spectral evidence for incorporation of the biotin label. Theoretical evidence for presence of surface protein characteristics was determined from protein primary sequences using established tools: the number of predicted TM domains was obtained from THMM2 [41] via PlasmoDB.org (*P*. *vivax* P01 v.31), presence of a signal peptide was predicted by SignalP version 4.1 [42] (http://www.cbs.dtu.dk/services/SignalP/) and presence of a glycosylphosphatidylinositol (GPI) anchor was predicted using PredGPI [43] (http://gpcr2.biocomp.unibo.it/gpipe/index.htm). A protein was considered to have spectral evidence for labeling if a non-degenerate component peptide displaying the addition of the biotin tag was identified from at least one high-quality PSM (PeptideProphet probability corresponding to an FDR less than 1.0%). Non-specific binding to the streptavidin beads was assessed by comparing the VK247 labeled and unlabeled samples, which were split from the same sample and processed in parallel with or without labeling. In order to identify those proteins with the highest value as potentially surface-exposed targets based on the theoretical and experimental evidence, proteins were assigned priority tiers (1 being highest) as follows: 1) possessing predicted TM domain(s), signal peptide or GPI anchor *and* exhibiting spectral evidence of incorporation of the biotin tag; 2) exhibiting spectral evidence of incorporation of the biotin tag but lacking predicted TM domain(s), signal peptide or GPI anchor; 3) possessing predicted TM domain(s), signal peptide or GPI anchor but lacking spectral evidence of incorporation of the biotin tag; 4) lacking predicted TM domain(s), signal peptide or GPI anchor as well as lacking spectral evidence of incorporation of the biotin tag. Tiers one, two and three were considered high-quality candidate surface proteins. Proteins identified from fewer than two peptides and three PSMs in at least one sample were not assigned priority tiers.

## Results

### Proteomic analysis of *P*. *vivax* field isolate-derived salivary gland sporozoites

**Fig 1:**
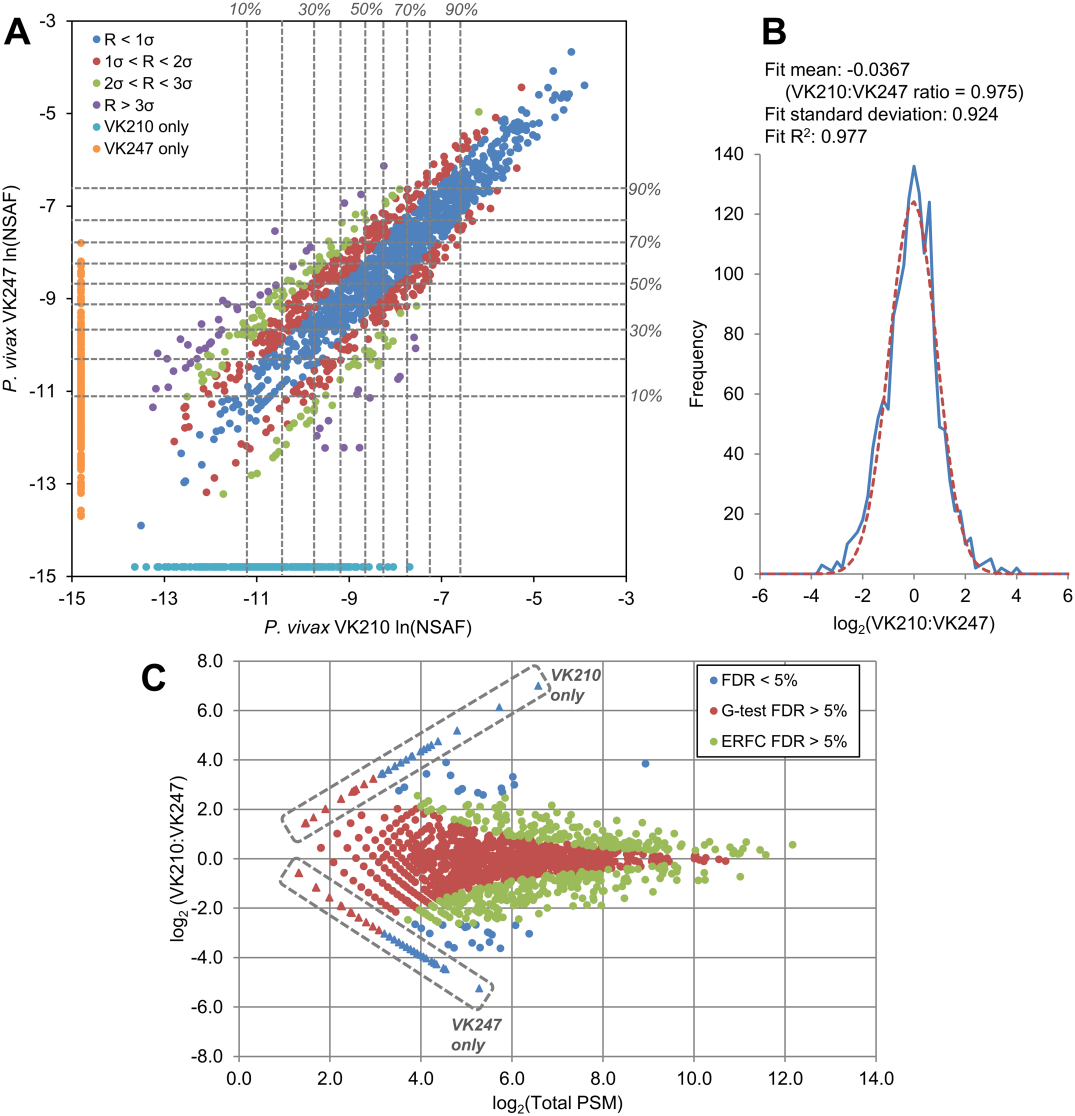
Quantitative comparison of protein expression between two *P*. *vivax* salivary gland sporozoite field isolates. (A) Protein abundances based on spectral counts were estimated using the normalized spectral abundance factor (NSAF). Each point represents the natural log-transformed NSAF value of a protein, comparing its ln(NSAF) value in either sample. Deciles of relative abundance within each sample are shown (dashed gray lines). For each protein observed in both of the two *P*. *vivax* salivary gland sporozoite samples, the natural log of the protein ratio of the NSAF values observed in the VK210 sample and the VK247 sample was calculated as ln(NASF)_VK210_-ln(NSAF)_VK247_. The population of these values produced a normal distribution centered near zero, corresponding to a mean ratio of 1:1 (S1 Fig). Proteins identified in both samples are color-coded to indicate the deviation of their log-transformed protein ratio *R* from the population mean as determined from the fit curve. Deviation from the mean was low at high abundances and increased with decreasing spectral counts. The cyan and orange points represent proteins identified in only one isolate or the other. (B) Protein ratios were calculated based on the adjusted and normalized spectral counts used to calculate the G statistic. The population of log-transformed protein ratios of proteins detected in both samples assumed a Gaussian distribution with a mean near zero. The mean and standard deviation from this distribution were used to calculate *p*-values using the complementary error function (ERFC). (C) The protein ratios of all proteins detected in either sample are plotted with respect to the sum of the adjusted and normalized PSM from both samples used to calculate the ratio for each protein. Points in red are proteins with that were not significant at a 5.0% false discovery rate (FDR) according to the G-test. Points in green are proteins with ratios that were not significant at a 5.0% FDR according to the ERFC. Points in blue are proteins ratios that were significant by both cut-offs. Points inside of dashed boxes represent proteins detected in only one sample or the other. Protein ratios were estimated for these proteins by increasing all spectral counts by one in order to give all proteins non-zero values.

MS-based proteomics was used to survey the proteins present in *P*. *vivax* salivary gland sporozoites. Two independent sporozoite samples were obtained from mosquitoes fed on blood obtained from volunteers who presented with clinical malaria at treatment centers in Thailand. Peptide spectrum matches were analyzed using the Trans-Proteomic Pipeline [24]. Proteins identified with scores corresponding to an FDR less than 1.0% were reported. A total of 1711 *P*. *vivax* proteins were identified from 3.5 × 10^6^ sporozoites bearing the VK210 haplotype of circumsporozoite protein (CSP), of which 1492 (87.2%) were identified by at least two non-degenerate peptides. A total of 1747 *P*. *vivax* proteins were identified from 4.5 × 10^6^ sporozoites bearing the VK247 CSP haplotype, of which 1572 (90.0%) were identified by at least two non-degenerate peptides. A combined total of 1970 *P*. *vivax* proteins were identified from the two samples, of which 1733 (88.0%) were identified from at least two non-degenerate peptides in at least one of the samples. Of the combined 1970 *P*. *vivax* proteins identified, 1488 (75.5%) were identified in both samples (S2 Table). Label-free protein quantification based on spectral counts was used to compare protein abundance between the two samples. NSAF, a technique that normalizes spectral counts for protein length and sample complexity, was used to compare relative protein abundance within and between the two samples, while protein abundance ratios between the two samples were tested for significance using the G-test as well as information extracted from the normal distribution of protein ratios. Comparing the protein abundances showed largely similar protein composition and protein abundance (Fig 1). The proteins identified in both samples included all of the proteins in the top quartile of abundance in each sample and 968 of 983 proteins (98.5%) in the upper half of abundance in each sample. Furthermore, 218 of 223 (97.8%) of the proteins unique to the VK210 sample were in the lower half of abundance, with 155 (69.5%) in the bottom quartile. Likewise, of the proteins unique to the VK247 sample, 249 of 259 (96.1%) were in the lower half of abundance, with 192 (74.1%) in the bottom quartile. These results suggest that differences in proteins detected between the two samples arose primarily from technical issues affecting limit-of-detection rather than unique protein expression in one sample or the other. Likewise, observed differences in relative protein abundance observed between the two samples were likely predominantly technical in origin rather than biological. The populations of ln(NSAF) values from the two field isolate samples could be fitted with Gaussian curves with similar means and variance, and the population of log-transformed abundance ratios for proteins detected in both samples assumed a normal distribution with a mean near zero, i.e., a protein ratio of essentially 1:1 (S1 Fig). Fitting the population of ratios to a Gaussian allowed measurement of the deviation from the mean of 1:1, which was low (less than one standard deviation) for high-abundance proteins and generally increased at lower protein abundances (Fig 1A), a known phenomenon of spectral counting [39, 44]. To identify proteins with significantly different abundances between the two samples, a likelihood ratio test (G-test) was applied to the protein ratios obtained from comparing spectral counts of each protein as observed in the two samples [40]. All spectral counts were increased by 1 in order to obtain ratios for proteins observed in only one sample [40]. Additionally, a Gaussian curve was fit to the distribution of log-transformed abundance ratios for proteins observed in both samples (Fig 1B) and the complementary error function was used to obtain a *p*-value indicating the probability that the normal distribution was more extreme than any give protein ratio. Combining these two tests identified protein ratios that deviated significantly from the mean while accounting for the increased quantification error at low spectral counts (Fig 1C). After correcting for multiple hypothesis testing by the Benjamini-Hochberg procedure, 119 proteins were identified with *p*-values corresponding to an FDR less than 5.0% by both methods. Of these, 35 were identified in both samples (2.4% of all proteins identified in both samples) and 84 were identified only in one sample or the other (17% of all proteins identified in only one of the two samples; S2 Table).

**Fig 2:**
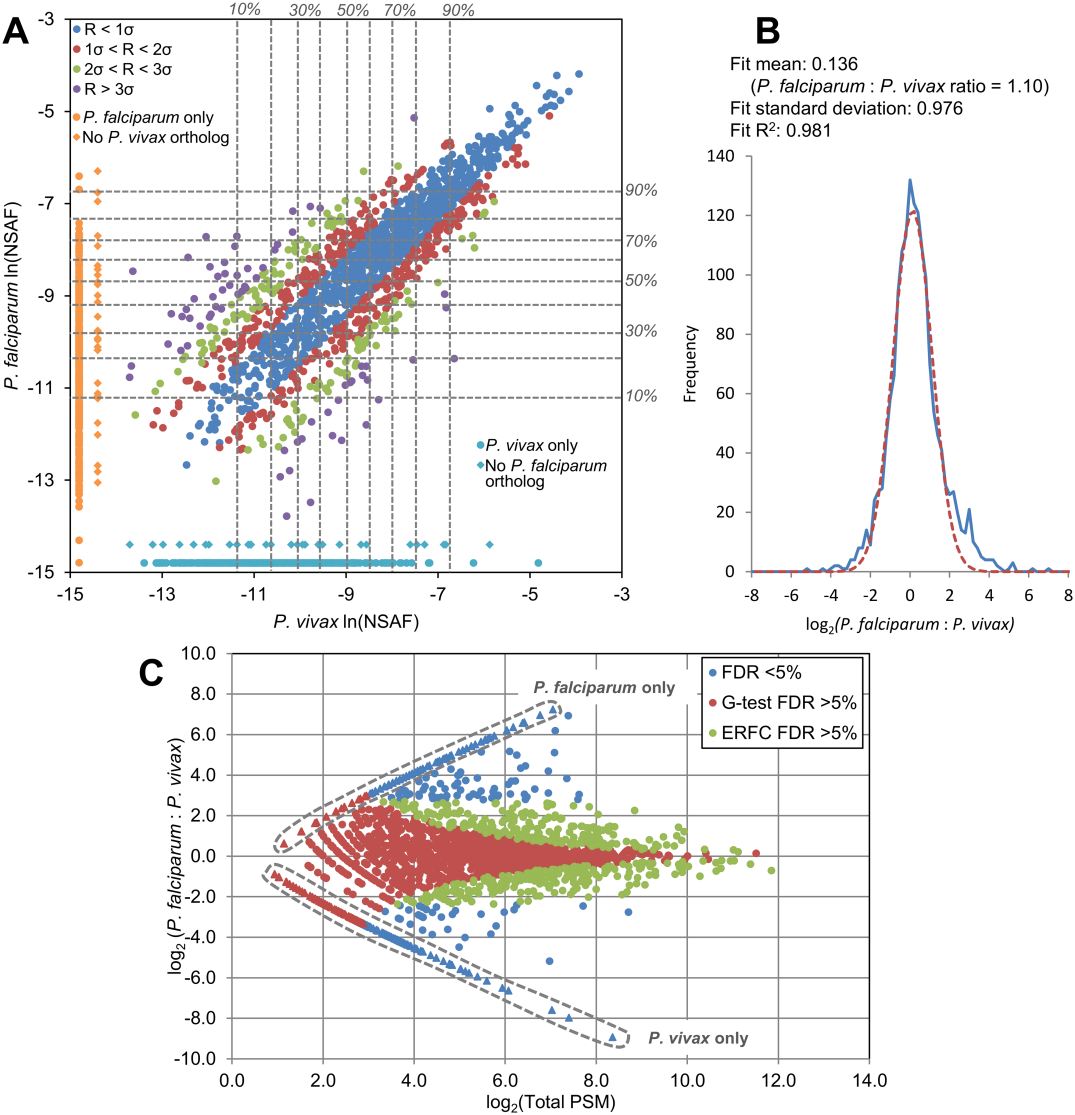
Quantitative comparison of protein expression between *P*. *vivax* and *P*. *falciparum* salivary gland sporozoites. (A) Protein abundances based on spectral counts were estimated using the normalized spectral abundance factor (NSAF). Each point represents the natural log-transformed NSAF value of a protein, comparing its ln(NSAF) value in either sample. *P*. *vivax* ln(NSAF) values are the average of the values observed in the two field isolate samples. Deciles of relative abundance within each sample are shown (dashed gray lines). For each protein with orthologs detected in both *P*. *falciparum* and *P*. *vivax* salivary gland sporozoites, the natural log of the protein ratio of the NSAF values observed in the *P*. *falciparum* sample and the *P*. *vivax* sample was calculated as ln(NASF)_*P*.*falciparum*_-ln(NSAF)_*P*.*vivax*_. The population of these values produced a normal distribution centered near zero, corresponding to a mean ratio of 1:1 (S2 Fig). Protein orthologs detected in both species are color-coded to indicate the deviation of their log-transformed protein ratio *R* from the population mean as determined from the fit curve. Deviation from the mean was low at high abundances and increased with decreasing spectral counts. The cyan and orange points represent protein orthologs identified in only one species or the other. Diamond points represent proteins with no ortholog in the other species. (B) Protein ratios were calculated based on the adjusted and normalized spectral counts used to calculate the G statistic. The population of log-transformed protein ratios of proteins detected in both samples assumed a Gaussian distribution with a mean near zero. The mean and standard deviation from this distribution were used to calculate *p*-values using the complementary error function (ERFC). (C) The protein ratios of all protein orthologs detected in either species are plotted with respect to the sum of the adjusted and normalized PSM from both samples used to calculate the ratio for each protein. Points in red are proteins with that were not significant at a 5.0% false discovery rate (FDR) according to the G-test. Points in green are proteins with ratios that were not significant at a 5.0% FDR according to the ERFC. Points in blue are proteins ratios that were significant by both cut-offs. Points inside of dashed boxes represent protein orthologs detected in only one species or the other. Protein ratios were estimated for these proteins by increasing all spectral counts by one in order to give all proteins non-zero values.

In order to compare salivary gland sporozoite proteomes of *P*. *vivax* and *P*. *falciparum*, a previously published proteomic analysis of *P*. *falciparum* salivary gland sporozoites [19] was re-analyzed with the same informatics pipeline used here, identifying 2010 proteins, of which 1798 (89.5%) were identified by at least two peptides (S3 Table). The same quantitative approach described above was used to compare the relative abundance of protein orthologs between the two species. The spectral counting methods were expected to be less accurate when comparing orthologs between species than when comparing the same proteins detected in different samples of the same species because, all else being equal, two protein orthologs with sufficiently different sequences could produce different numbers of PSMs due to differences in the number of tryptic peptides produced and the detectability of these peptides by LC-MS determined by sequence-specific chemical properties. Nonetheless, there was a large overlap in both protein detection and relative protein abundance between protein orthologs detected in the *P*. *vivax* and *P*. *falciparum* samples (Fig 2). Of the all the proteins detected in either of the *P*. *vivax* samples or the *P*. *falciparum* sample, 2314 had annotated orthologs in both *P*. *falciparum* and *P*. *vivax*, and 1609 of these (69.5%) were detected in the sporozoite samples of both species analyzed here. As with the comparison between the two *P*. *vivax* samples, the population of log-transformed ratios of proteins identified in both *P*. *vivax* and *P*. *falciparum* had a mean near 1:1 with little deviation from the mean among the high-abundance proteins and increasing deviation at low spectral counts. Most of the protein orthologs identified in one species and not the other were low-abundance proteins. Of the 332 orthologs not detected in the *P*. *falciparum* sample, 300 (90.4%) were in the lower half of abundance and 189 (56.9%) were in the bottom quartile of abundance. Of the 373 orthologs not detected in the *P*. *vivax* samples, 325 (87.1%) were in the lower half of abundance and 224 (60.1%) were in the bottom quartile of abundance (S3 Table, Fig 2A). The most highly abundant proteins detected in the *P*. *vivax* sporozoites were also highly abundant in *P*. *falciparum* sporozoites, including several with critical roles in invasion, e.g., CSP, thrombospondin-related anonymous protein (TRAP; PVP01_1218700, PF3D7_1335900), gamete egress and sporozoite traversal protein (GEST; PVP01_1258000, PF3D7_1449000), cell traversal protein for ookinetes and sporozoites (CelTOS; PVP01_1435400, PF3D7_1216600), apical membrane antigen 1 (AMA1; PVP01_0934200, PF3D7_1133400), sporozoite invasion-associated protein 1 (SIAP1; PVP01_0307900, PF3D7_0408600), and sporozoite protein essential for cell traversal (SPECT1 PVP01_1212300, PF3D7_1342500) (Table 1). Even so, a number of high-abundance proteins were identified that were of significantly higher abundance in one species than the other (S3 Table). For example, PVP01_0314600 (conserved *Plasmodium* protein, unknown function) was in the top decile of abundance in both *P*. *vivax* sporozoite samples, while its syntenic ortholog PF3D7_0718900 (conserved *Plasmodium* protein, unknown function) was not detected in the *P*. *falciparum* sporozoite sample, or for that matter, in any of the *P*. *falciparum* proteomics datasets on PlasmoDB spanning the entire *P*. *falciparum* lifecycle. In the *P*. *falciparum* sporozoite sample, two conserved *Plasmodium* proteins of unknown function, PF3D7_0215200 and PF3D7_0410500, were in the top decile of protein abundance but not detected at all in either *P*. *vivax* sample. Both proteins are up-regulated in *P*. *falciparum* salivary gland sporozoites based on transcriptomic and proteomic data compiled at PlasmoDB.org. In addition to differentially expressed orthologs, a number of identified proteins had no orthologs in the other species compared. Of the combined 1970 *P*. *vivax* proteins identified, 29 (1.47%) had no *P*. *falciparum* ortholog. These included three proteins annotated as PIR proteins (*Plasmodium* interspersed repeats, species-specific immunovariant proteins [34, 45]) and 17 unannotated proteins (i.e., “conserved *Plasmodium* protein, unknown function”). The most abundant *P*. *vivax* protein with no *P*. *falciparum* ortholog identified in the samples was a putative 6-Cys domain protein (PVP01_0303900). This protein was in the top decile of abundance in both *P*. *vivax* samples, and is putatively surface-exposed on salivary gland sporozoites (discussed below).

**Table 1:** Highly abundant proteins identified in *P*. *vivax* salivary gland sporozoites.

Abundance rank (of 1970)^a^	Gene ID	Protein	Description	*P*. *falciparum* ortholog^b^	*P*. *falciparum* abundance rank (of 2010)^c^
1	PVP01_0905800	H4	histone H4, putative	PF3D7_1105000	1
2	PVP01_1218700	TRAP	thrombospondin-related anonymous protein, putative	PF3D7_1335900	4
3	PVP01_0518800	HSP20	small heat shock protein HSP20, putative	PF3D7_0816500	8
4	PVP01_0808400	null	tubulin beta chain, putative	PF3D7_1008700	2
5	PVP01_1244000	GAPDH	glyceraldehyde-3-phosphate dehydrogenase, putative	PF3D7_1462800	11
6	PVP01_0835600	CSP	circumsporozoite (CS) protein	PF3D7_0304600	6
7	PVP01_1463200	ACT1	actin	PF3D7_1246200	9
8	PVP01_0905900	H2B	histone 2B, putative	PF3D7_1105100	5
9	PVP01_0717700	TrxL1	thioredoxin-like protein 1, putative	PF3D7_0919300	17
10	PVP01_1114800, PVP01_1114900	null	elongation factor 1-alpha, putative	PF3D7_1357000, PF3D7_1357100	7
11	PVP01_1258000	GEST	gamete egress and sporozoite traversal protein, putative	PF3D7_1449000	12
12	PVP01_0517100	14-3-3I	14-3-3 protein, putative	PF3D7_0818200	14
13	PVP01_1311000	PfpUB	polyubiquitin 5, putative	PF3D7_1211800	N/A^d^
14	PVP01_1030500	ADF1	actin-depolymerizing factor 1, putative	PF3D7_0503400	3
15	PVP01_0934200	AMA1	apical membrane antigen 1	PF3D7_1133400	20
16	PVP01_1020600	PNP	purine nucleoside phosphorylase, putative	PF3D7_0513300	29
17	PVP01_1435400	CelTOS	cell traversal protein for ookinetes and sporozoites	PF3D7_1216600	91
18	PVP01_0307900	SIAP1	sporozoite invasion-associated protein 1, putative	PF3D7_0408600	23
19	PVP01_1262200	FBPA	fructose 1,6-bisphosphate aldolase, putative	PF3D7_1444800	25
20	PVP01_0702100	null	alpha tubulin 1, putative	PF3D7_0903700	15
21	PVP01_1008000	IMC1g	inner membrane complex protein 1g, putative	PF3D7_0525800	60
22	PVP01_1212300	SPECT1	sporozoite protein essential for cell traversal, putative	PF3D7_1342500	68
23	PVP01_1425700	null	conserved Plasmodium protein, unknown function	PF3D7_0814600	24
24	PVP01_1444500	HAD2	haloacid dehalogenase-like hydrolase, putative	PF3D7_1226300	97
25	PVP01_0920700	PGM1	phosphoglycerate mutase, putative	PF3D7_1120100	26
26	PVP01_0938800	SPELD	sporozoite surface protein essential for liver stage development, putative	PF3D7_1137800	10
27	PVP01_0918300	RAN	GTP-binding nuclear protein RAN/TC4, putative	PF3D7_1117700	61
28	PVP01_0505600	GAMA	GPI-anchored micronemal antigen	PF3D7_0828800	79
29	PVP01_1229700	LDH	L-lactate dehydrogenase	PF3D7_1324900	21
30	PVP01_0728100	null	G2 protein, putative	PF3D7_0929600	30
31	PVP01_1306500	null	conserved Plasmodium protein, unknown function	PF3D7_1207400	37
32	PVP01_0819300	H2A.Z	histone H2A.Z, putative	PF3D7_0320900	39
33	PVP01_1454700	null	p25-alpha family protein, putative	PF3D7_1236600	56
34	PVP01_1212200	MyoA	myosin A, putative	PF3D7_1342600	18
35	PVP01_1411700	null	RNA-binding protein, putative	PF3D7_1310700	46

^a)^ Proteins ranked in order of decreasing abundance using the normalized spectral abundance factor.

^b)^ Syntenic orthologs in *P*. *falciparum* as annotated in PlasmoDB.

^c)^ Protein abundance ranks from proteomic analysis of *P*. *falciparum* salivary gland sporozoites [19] re-analyzed with the same software and parameters used here.

^d)^ Polyubiquitin (PF3D7_1211800) has extensive regions of identical sequence with ubiquitin-60S ribosomal protein L40 (PF3D7_1365900). In the *P*. *falciparum* sample, all peptides identifying polyubiquitin were shared with L40. Peptides specific to L40 but none specific to polyubiquitin were identified, so by the parsimony rules of ProteinProphet, all identifying spectra were assigned to L40 (giving it an abundance rank of 19) and polyubiquitin was not considered identified. Non-degenerate peptides unique to both orthologs were identified in the *P*. *vivax* samples (S2 and S3 Tables).

Mass spectra were searched against the *P*. *vivax* P01 reference proteome [34] (PlasmoDB v.31[29]). Current high-throughput MS approaches require a precise knowledge of the genome of the organism under study; a protein can only be identified if its exact sequence is contained in the database against which the mass spectra are searched. Because the samples were obtained from field isolates and not laboratory strains, they were expected to contain protein sequence polymorphisms that would not be represented in the reference proteome. In order to increase the likelihood of identifying isolate-specific polymorphisms, the *P*. *vivax* protein database against which the mass spectra were searched was augmented with potential polymorphisms obtained from genomic and transcriptomic analyses of Thai *P*. *vivax* field isolates. A total of 13 RNA-seq and 19 DNA-seq data sets were aligned against the *P*. *vivax* Sal1 reference genome and a reference proteome was generated containing any protein with an amino acid sequence differing from the reference. Only 22% of the proteins in the reference proteome had completely conserved sequences across all 33 datasets (the 32 field isolates plus the reference genome). Over 50% of the proteins had four or more unique amino acid sequences arising from various combinations of sequence polymorphisms, and 10% had 15 or more unique sequences. One protein, RNA pseudouridylate synthase (PVX_080660) had a unique sequence in all 33 genomes aligned (S4 Table). These *P*. *vivax* Sal-1 variants and the *P*. *vivax* Sal-1 reference proteome were appended to the *P*. *vivax* P01 reference proteome and used to identify polymorphisms in the analyzed samples. A total of 301 identified *P*. *vivax* proteins contained polymorphisms that were not present in the *P*. *vivax* P01 reference proteome (S5 Table). The four identified *P*. *vivax* salivary gland sporozoite proteins exhibiting the most polymorphisms not present in the *P*. *vivax* P01 reference proteome were surface proteins: AMA1 (PVP01_0934200), TRAP-like protein (TLP; PVP01_1132600), TRAP (PVP01_1218700) and GPI-anchored micronemal protein (GAMA; PVP01_0505600). Each of these proteins also exhibited a high degree of polymorphism in the compared field isolate genomes (95^th^, 89^th^, 98^th^, and 97^th^ percentiles, respectively, of the number of unique protein sequences arising from polymorphisms among the compared genomes). Except for seven proteins identified from a single peptide, all of these polymorphism-bearing proteins could be detected without the additional knowledge of polymorphisms obtained from the field isolates. However, failing to detect peptides would have led to increased errors in protein quantification by spectral counts. Furthermore, knowledge of non-synonymous substitutions in the *P*. *vivax* genome was critical to accurately characterizing proteins detected in the samples. For example, the *P*. *vivax* haplotype designations VK210 and VK247 are based on differences in the sequence of the repeat region of CSP. The VK210 haplotype bears tandem repeats of the sequence GDRA(D/A)GQPA, while the VK247 haplotype bears tandem repeats of the sequence ANGA(G/D)(N/D)QPG. In the VK247 whole proteome analyzed here, the repeat region of CSP was poorly detected due to a lack of Lys or Arg residues that would result in tryptic peptides. However, hundreds of PSMs identified a tryptic peptide at the C-terminal end of the tandem repeat region which is distinct in VK247 [35], and no independent evidence was observed for VK210-specific peptides (S3 Fig, S5 Table). Conversely, in the VK210 sample, peptides covering the entire CSP tandem repeat region were identified from hundreds of PSMs, owing to the presence of regularly interspersed Arg tryptic cleavage sites. Interestingly, the VK210 sample appeared to contain a mixed infection of at least two distinct field isolates. The same discriminating peptide at the C-terminal end of the tandem repeat region was identified by hundreds of PSMs for semi-tryptic fragments of various lengths containing the VK210-specific sequence found in the *P*. *vivax* P01 reference proteome. However, a semi-tryptic variant of the peptide found in the *P*. *vivax* Sal-1 version of CSP was also identified, as were semi-tryptic peptides matching portions of the VK247 version of the peptide. There was not enough independent evidence to determine if the VK247 haplotype was present in the sample (S3 Fig, S5 Table). Evidence for a mixed infection was also found in TRAP. Seven sequence polymorphisms not present in the *P*. *vivax* P01 reference proteome were identified in TRAP in the samples analyzed, four of which were present in the *P*. *vivax* Sal-1 reference proteome and three of which were only found in field isolates. As was observed for CSP, the VK247 sample appeared to contain a single haplotype of TRAP, while there were at least two haplotypes of TRAP in the VK210 sample (S4 Fig, S5 Table). In addition to accurate quantification and correct characterization of proteins, knowledge of sample-specific polymorphisms was critical to identifying post-translational modifications (discussed below).

### Post-translational modifications

**Fig 3:**
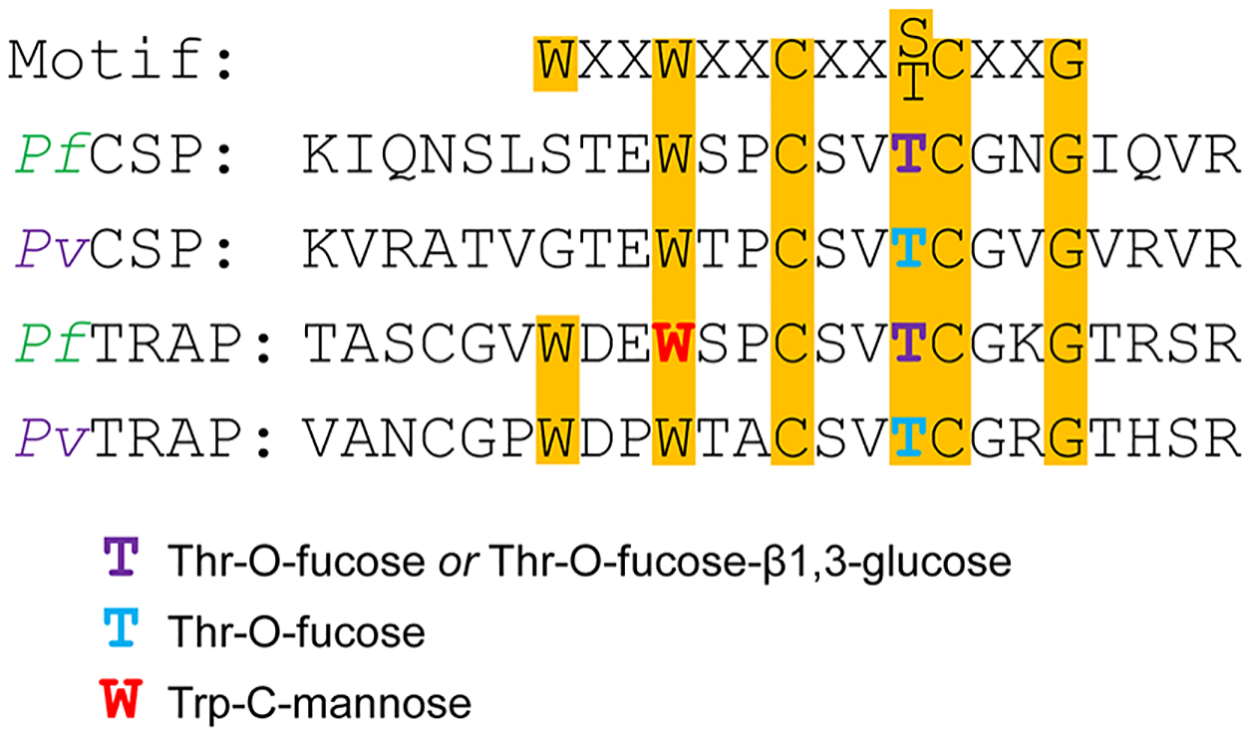
Glycosylation of CSP and TRAP in *P*. *vivax* and *P*. *falciparum* salivary gland sporozoites. The glycosylated portions of the conserved thrombospondin repeat (TSR) domains of *P*. *falciparum* and *P*. *vivax* CSP and TRAP are aligned. The conserved glycosylation motif is highlighted. Residues that are putatively glycosylated according to the MS evidence are colored as shown in the legend. Information on glycosylation of *P*. *falciparum* is from [20]. Only TRAP in *P*. *falciparum* salivary gland sporozoites exhibited evidence for C-mannosylation. Both CSP and TRAP exhibited evidence for O-fucosylation in both species. In *P*. *falciparum*, a portion of CSP and TRAP also showed evidence for modification with a fucose-glucose disaccharide. No evidence for the disaccharide was observed in the *P*. *vivax* samples.

It was recently shown that CSP and TRAP are glycosylated in *P*. *falciparum* salivary gland sporozoites [20]. Here we report that these proteins are similarly modified in *P*. *vivax* sporozoites. The motif CX_2-3_(S/T)CXXG in thrombospondin repeat (TSR) domains can be modified with an O-linked fucose at the Ser/Thr [46], and this fucose can be further modified with glucose to produce a β1,3-linked disaccharide [47, 48]. Additionally, the WXXW and WXXC motifs of TSR domains can be modified with a C-linked mannose at Trp [49, 50]. These potential glycosylation motifs are present in the TSR domains of both CSP and TRAP in all *Plasmodium* species. The TSR domain of *P*. *vivax* CSP contains the tryptic peptide ATVGTEWTPCSVTCGVGVR with potential O-fucosylation and C-mannosylation sites. Modification of the peptide with O-linked glycans could not be directly detected by the spectral search engines due to the fact that O-linked glycans are highly labile in the gas phase [51, 52] and are lost during collision-induced dissociation (CID) used to generate the identifying fragment spectra. However, as was previously demonstrated with *P*. *falciparum* salivary gland sporozoites [20], it was possible to infer the presence of the O-linked glycan through manual interpretation of the mass spectra (S5 and S6 Figs). The analysis showed that this peptide was modified with a mass matching that of an O-linked deoxyhexose. No evidence for C-mannose was observed (Fig 3). While neither the identity of the deoxyhexose nor its attachment site in the peptide could be determined from the data, we presume it to be a fucose attached to the C-terminal Thr based on knowledge of the sugars and enzymes present in *Plasmodium* [53], TSR domains in other species, and the fact that this residue has been shown to be O-fucosylated in crystal structures of *Pf*CSP expressed in mammalian cells [54]. Evidence for O-fucosylation of CSP was observed in both samples. Based on the signal intensity of the LC peaks, it appears that the majority of CSP (~90%) was glycosylated while a portion was unmodified (S5 and S6 Figs). In *P*. *falciparum* sporozoites, the majority of CSP was also observed to be modified with a single deoxyhexose while a small portion was unmodified, though some CSP was also observed to be further modified with an additional hexose, consistent with O-linked fucose-β1,3-glucose [20]. No evidence for modification of CSP with a disaccharide was observed in these *P*. *vivax* sporozoite samples.

The TSR domain of *P*. *vivax* TRAP contains the tryptic peptide VANCGPWDPWTACSVTCGR which includes potential O-fucosylation and C-mannosylation motifs. Critically, the TRAP in the VK247 sample and some of the TRAP in the mixed-infection VK210 sample exhibited an Arg→Lys substitution at this peptide. Knowledge of this polymorphism was only obtained from the field isolate genomes, so lacking that data would have prevented detecting glycosylation in the samples bearing the substitution. As with CSP, TRAP was observed to be modified with a gas-phase labile modification (S7 and S8 Figs) which was presumed to be O-fucose attached at the C-terminal Thr, again based on the TSR motif as well as crystal structures of *Pv*TRAP and *Pf*TRAP expressed in mammalian cells [55]. C-mannosylation of the WDPWTAC sequence was not observed (Fig 1), even though in *P*. *falciparum* sporozoites the C-terminal Trp of WDEWSPC was modified with a mass matching that of hexose, likely C-mannose [20]. Based on chromatographic peak areas, virtually all TRAP in both samples was completely glycosylated (S7 and S8 Figs).

The MS data were further analyzed for evidence of protein phosphorylation, a reversible PTM that is often involved in signaling and control of cellular function. Proteomic analysis of phosphoproteins has been performed for asexual stages of *P*. *falciparum* [56–60] but not sporozoites. Typical phosphoproteomic analyses employ affinity techniques to enrich for phosphorylated peptides prior to LC-MS. While that approach was not feasible for this study due to the limited sample material available, it was still possible to detect the presence of this modification in proteins that were highly abundant and/or heavily modified in the samples. Evidence for phosphorylation was found for a total of 139 proteins in either or both of the samples (S6 and S7 Tables). Among the detected phosphoproteins with GO terms, the most prevalent functional class was proteins with DNA or RNA-binding activity (21.6% of the phosphoproteins) and the second most prevalent class was proteins with ATP activity, e.g., ATP binders, kinases and phosphatase (20.9% of the phosphoproteins). Also well-represented were components of the gliding machinery, including Myosin A (MyoA; PVP01_1212200), the glideosome-associated proteins GAP40 (PVP01_1018200), GAP45 (PVP01_1440900) and GAPM2 (PVP01_0532000), several inner membrane complex (IMC) proteins, and the calcium-dependent protein kinase CDPK1 (PVP01_0407500). The *P*. *falciparum* salivary gland sporozoite data were searched in the same fashion, identifying 91 phosphorylated proteins (S6 and S8 Tables). All but four of these had syntenic orthologs in *P*. *vivax*, and 48 of these (55%) were among the phosphoproteins identified from the *P*. *vivax* samples. The list of sporozoite phosphoproteins was compared against thirteen proteomic analyses of *P*. *falciparum* blood-stage parasites [16, 56–66] (including five analyses of phosphopeptides enriched from blood-stage parasites [56–60]) and three proteomic analyses of *P*. *vivax* blood-stage parasites [61, 67, 68] available on PlasmoDB.org. The majority (74%) of phosphoproteins identified from either *P*. *vivax* or *P*. *falciparum* sporozoites were also identified in phosphorylated form in *P*. *falciparum* blood stages (S6 Table). Table 2 lists 16 *P*. *vivax* sporozoite phosphoproteins whose orthologs were identified in *P*. *falciparum* blood stages but for which no evidence of phosphorylation was observed, potentially representing sporozoite-specific phosphorylation. Table 3 lists 18 *P*. *vivax* sporozoite phosphoproteins that were not detected at all (either phosphorylated or unphosphorylated) in proteomic analyses of *P*. *falciparum* and *P*. *vivax* blood stages, representing known and potentially novel proteins specific to the sporozoite stage.

**Table 2:** Proteins phosphorylated in sporozoites but not in blood stages^a^.

Gene ID	*P*. *falciparum* ortholog	Protein	Protein description	Phospho in *P*. *falciparum* sporozoites?^b^	Upregulated in sporozoites?^c^	Annotated function^d^
PVP01_1310200	PF3D7_1211000	-	kinesin-7, putative	YES	YES	ATP binding, ATPase activity, microtubule motor activity
PVP01_0836200	PF3D7_0304000	IMC1a	inner membrane complex protein 1a, putative	YES	-	-
PVP01_1128100	PF3D7_0621400	ALV7	inner membrane complex protein 1j, putative	YES	-	-
PVP01_1341000	PF3D7_1407700	-	conserved Plasmodium protein, unknown function	-	YES	-
PVP01_1225100	PF3D7_1329400	-	AMP deaminase, putative	-	-	AMP deaminase activity
PVP01_0211800	PF3D7_0102900	-	aspartate—tRNA ligase, putative	-	-	ATP binding, aspartate-tRNA ligase activity, nucleic acid binding
PVP01_0215600	PF3D7_0729900	-	dynein heavy chain, putative	-	-	ATP binding, ATPase activity, microtubule motor activity
PVP01_0702400	PF3D7_0904000	-	GTPase-activating protein, putative	-	-	Rab GTPase activator activity
PVP01_0420400	PF3D7_0204700	HT	hexose transporter	-	-	substrate-specific transmembrane transporter activity
PVP01_1212000	PF3D7_1342800	PEPCK	phosphoenolpyruvate carboxykinase, putative	-	-	ATP binding, phosphoenolpyruvate carboxykinase (ATP) activity
PVP01_0505000	PF3D7_0829400	-	prolyl 4-hydroxylase subunit alpha, putative	-	-	L-ascorbic acid binding, iron ion binding, oxidoreductase activity, acting on paired donors, with incorporation or reduction of molecular oxygen
PVP01_1102300	PF3D7_1369700	-	U2 small nuclear ribonucleoprotein A', putative	-	-	protein binding
PVP01_1249600	PF3D7_1457300	-	conserved Plasmodium protein, unknown function	-	-	binding
PVP01_0811600	PF3D7_1011500	-	conserved Plasmodium protein, unknown function	-	-	-
PVP01_1454300	PF3D7_1236200	-	conserved Plasmodium protein, unknown function	-	-	protein binding
PVP01_1411500	PF3D7_1310500	-	conserved protein, unknown function	-	-	carbohydrate binding

^a)^ A combined total of 139 *P*. *vivax* proteins were identified with evidence for phosphorylation from proteomic analyses of two different salivary gland sporozoite samples. Presented in this table are the 16 proteins whose orthologs were not detected in phosphorylated form in any of six published phosphoproteomic analyses of *P*. *falciparum* blood stages [56–60] available from PlasmoDB and yet were still detectable in *P*. *falciparum* blood stages in any of the 13 proteomic analyses [16, 56–66] available from PlasmoDB. The complete list of detected phosphoproteins is provided in S6 Table, and the complete list of detected phosphopeptides is provided in S7 and S8 Tables.

^b)^ “YES” indicates that the protein was also detected with phosphorylation in the re-analysis of the previously published *P*. *falciparum* salivary gland sporozoite proteome presented here.

^c)^ “YES” indicates that the protein is annotated as up-regulated in salivary gland sporozoites in PlasmoDB, either identified as up-regulated in the Winzeler OPI gene expression data or identified as a Sporozoite Conserved Orthologous Transcript (SCOT).

^d)^ Protein functions are annotated and/or predicted GO terms obtained from PlasmoDB v.32.

**Table 3:** Phosphorylated sporozoite-specific proteins^a^.

Gene ID	*P*. *falciparum* ortholog	Protein	Protein description	Phospho in *P*. *falciparum* sporozoites?^b^	Upregulated in sporozoites?^c^	Annotated function^d^
PVP01_1015000	PF3D7_0518900	-	conserved Plasmodium protein, unknown function	YES	YES	-
PVP01_1439700	PF3D7_1221400	IMC1h	inner membrane complex protein 1h, putative	YES	-	-
PVP01_0518800	PF3D7_0816500	HSP20	small heat shock protein HSP20, putative	YES	-	-
PVP01_1427900	PF3D7_0812300	SSP3	sporozoite surface protein 3, putative	YES	-	-
PVP01_0938800	PF3D7_1137800	SPELD	sporozoite surface protein essential for liver stage development, putative	YES	-	-
PVP01_0945700	PF3D7_1145000	-	conserved Plasmodium protein, unknown function	YES	-	ATP binding, actin binding, calmodulin binding, motor activity, sequence-specific DNA binding, sequence-specific DNA binding transcription factor activity
PVP01_0415300	PF3D7_0209500	-	conserved Plasmodium protein, unknown function	YES	-	GTP binding, GTPase activity, translation initiation factor activity
PVP01_1032700	PF3D7_0502300	-	conserved Plasmodium protein, unknown function	YES	-	-
PVP01_1259500	PF3D7_1447500	IMC20	conserved Plasmodium protein, unknown function	YES	-	-
PVP01_1432200	PF3D7_1213400	-	conserved Plasmodium protein, unknown function	YES	-	-
PVP01_1218700	PF3D7_1335900	TRAP	thrombospondin-related anonymous protein, putative	-	YES	host cell surface receptor binding
PVP01_1448500	PF3D7_1230300	SPM2	subpellicular microtubule protein 2, putative	-	-	transferase activity
PVP01_1132600	PF3D7_0616500	TLP	TRAP-like protein, putative	-	-	-
PVP01_1124700	PF3D7_0624800	-	conserved Plasmodium protein, unknown function	-	-	ATP binding
PVP01_0813500	PF3D7_1013400	-	conserved Plasmodium protein, unknown function	-	-	ATP binding
PVP01_1425600	PF3D7_0814700	null	conserved Plasmodium protein, unknown function	-	-	-
PVP01_0947000	-	-	conserved Plasmodium protein, unknown function	-	-	-
PVP01_0609000	-	-	conserved Plasmodium protein, unknown function	-	-	-

^a)^ A combined total of 139 *P*. *vivax* proteins were identified with evidence for phosphorylation from proteomic analyses of two different salivary gland sporozoite samples. Presented in this table are the 18 proteins that were not detected in any of the of the 13 proteomic analyses of blood stage *P*. *falciparum* [16, 56–66] available from PlasmoDB.org. The complete list of detected phosphoproteins is provided in S6 Table, and the complete list of detected phosphopeptides is provided in S7 and S8 Tables.

^b)^ “YES” indicates that the protein was also detected with phosphorylation in the re-analysis of the previously published *P*. *falciparum* salivary gland sporozoite proteome presented here.

^c)^ “YES” indicates that the protein is annotated as up-regulated in salivary gland sporozoites in PlasmoDB.org, either identified as up-regulated in the Winzeler OPI gene expression data or identified as a Sporozoite Conserved Orthologous Transcript (SCOT).

^d)^ Protein functions are annotated and/or predicted GO terms obtained from PlasmoDB v.32.

### Identification of surface-exposed proteins

In order to identify surface-exposed proteins on *P*. *vivax* salivary gland sporozoites, a chemical labeling approach was employed based on the recent analyses of the *P*. *falciparum* salivary gland sporozoite surface proteome [19, 20]. Live sporozoites were labeled with a membrane-impermeable, amine-reactive tag that covalently labeled solvent-exposed lysines with a biotin tag. The parasites were then lysed and labeled proteins were recovered with streptavidin beads. Two parasite samples were analyzed, one containing 2 × 10^6^ sporozoites bearing the VK210 CSP haplotype and one containing 1.8 × 10^7^ sporozoites bearing the VK247 CSP haplotype. The VK247 sample was split in two and half was left unlabeled in order to assess non-specific binding. A total of 90 *Plasmodium* proteins were identified from the VK210 sample, of which 61 (68%) were identified from two or more peptides, and 221 *Plasmodium* proteins were identified from the labeled VK247 sample, of which 147 (67%) were identified from two or more peptides. The combined samples identified 239 *Plasmodium* proteins, of which 72 (30%) were identified in both samples. The 129 proteins identified from two or more peptides and three or more PSM in at least one sample were taken for further analysis. Some proteins could be seen to exhibit direct evidence for incorporation of the biotin label in the identifying mass spectra. Absence of spectral evidence for labeling does not mean that the protein was not labeled [69], but observing labeling in highly abundant sporozoite surface proteins such as CSP and TRAP provides evidence that the labeling and enrichment protocol successfully identified surface-exposed proteins. The non-specific binding was very low—only eight *Plasmodium* proteins were identified from the unlabeled sporozoites (five identified from two or more peptides) compared to the 221 *Plasmodium* proteins identified from an equal number of labeled sporozoites from the same sample. The eight *Plasmodium* proteins in the control were identified by 49 PSMs, more than 30-fold fewer than the 1604 PSMs obtained from the labeled sample (S9 Table). The labeled and unlabeled VK247 sporozoites were split from the same batch of purified sporozoites and, except for the labeling steps, were processed identically in parallel along with the labeled VK210 sample, including lysis, capture on magnetic biotin beads, washes, elution, SDS-PAGE and in-gel digestion, and all three samples were analyzed by LC-MS one after the other on the same column. As such, the raw number of spectral counts gives the best estimate of relative abundance when comparing the relative abundance of a protein identified in both the labeled and unlabeled VK247 samples. Seven of the eight proteins identified from the unlabeled control were also identified in the labeled sample. Although there was insufficient data to assess statistically significant enrichment of labeled versus unlabeled proteins, all seven proteins were at least two-fold more abundant in the labeled sample based on the number of PSM. Proteins identified in the unlabeled control included the known sporozoite surface proteins TRAP and sporozoite surface protein essential for liver stage development (SPELD; PVP01_0938800) [70], as well as actin (PVP01_1463200), which has been detected on the surface of ookinetes [71]. These proteins exhibited direct evidence from the identifying mass spectra that they had been labeled with the biotin tag the labeled samples. They were also among the most abundant proteins in the sporozoite proteome (top 2%), so their presence among non-specifically binding proteins is not surprising. Given the above, the contribution of non-specific binding to the proteins identified in both samples was assumed to be minimal. In order to rule out low-confidence identifications, only proteins identified from two or more peptides and three or more PSM were taken for further analysis.

Although the biotin tag for surface labeling is putatively membrane-impermeable [72], based on previous work, some portion of sporozoites were assumed to have compromised plasma membranes, resulting in labeling of intracellular proteins [20, 73]. Therefore, combined theoretical and experimental evidence were used to identify the strongest candidates for surface-exposed proteins from among all those identified by the surface protein enrichment strategy. Proteins that were identified with high confidence as described above were assigned a priority tier (1 being highest) as follows: Tier 1) possessing predicted transmembrane (TM) domain(s), signal peptide or glycophosphatidylinositol (GPI) anchor *and* exhibiting spectral evidence of incorporation of the biotin tag; 2) exhibiting spectral evidence of incorporation of the biotin tag but lacking predicted TM domain(s), signal peptide or GPI anchor; 3) possessing predicted TM domain(s), signal peptide or GPI anchor but lacking spectral evidence of incorporation of the biotin tag; 4) lacking predicted TM domain(s), signal peptide or GPI anchor as well as lacking spectral evidence of incorporation of the biotin tag. These criteria produced a list of 36 high-quality candidate surface proteins (Table 4). Of these, 31 orthologs were also detected by similar analyses of putatively surface-exposed proteins on *P*. *falciparum* [20] or *P*. *yoelii* [74] salivary gland sporozoites. Several of these are known to be secreted and/or surface-exposed on sporozoites, including CSP, TRAP, SPELD [70], GEST, sporozoite surface protein 3 (SSP3; PVP01_1427900), hexose transporter (HT; PVP01_0420400) and CelTOS.

**Table 4:** Putatively surface-exposed proteins on *P*. *vivax* salivary gland sporozoites.

Tier^a^	Gene ID	Protein	Protein Description	Samples ID'd^b^	*P*. *vivax* evidence^c^	*P*. *falciparum* evidence^d^	*P*. *yoelii* evidence^e^
1	PVP01_0835600	CSP	circumsporozoite (CS) protein	2	Labeled, Signal, GPI	Enriched, Labeled	Detected
1	PVP01_0938800	SPELD	sporozoite surface protein essential for liver stage development, putative	2	Labeled, TM	Enriched, Labeled	Detected
1	PVP01_1218700	TRAP	thrombospondin-related anonymous protein, putative	2	Labeled, TM, Signal	Enriched, Labeled	Detected
1	PVP01_1258000	GEST	gamete egress and sporozoite traversal protein, putative	1	Labeled, Signal	Detected	Detected
2	PVP01_1463200		actin	2	Labeled	Enriched, Labeled	Detected
2	PVP01_1227100		conserved Plasmodium protein, unknown function	2	Labeled	Detected	-
2	PVP01_0602700		conserved Plasmodium protein, unknown function	2	Labeled	Enriched	-
2	PVP01_1212200		myosin A, putative	2	Labeled	Enriched, Labeled	Detected
2	PVP01_1311000		polyubiquitin 5, putative	2	Labeled	Enriched	Detected
2	PVP01_0518800	HSP20	small heat shock protein HSP20, putative	2	Labeled	Enriched	-
2	PVP01_1268100	TPx1	thioredoxin peroxidase 1, putative	2	Labeled	Enriched	Detected
3	PVP01_0303900		6-cysteine protein, putative, pseudogene	2	Signal, GPI	NO ORTHOLOG	NO ORTHOLOG
3	PVP01_0621700	ADT	ADP/ATP transporter on adenylate translocase, putative	2	3 TMs	Enriched	-
3	PVP01_0934200	AMA1	apical membrane antigen 1	2	TM	Enriched	-
3	PVP01_1435400	CelTOS	cell traversal protein for ookinetes and sporozoites	2	TM	-	-
3	PVP01_0532000	GAPM2	glideosome associated protein with multiple membrane spans 2, putative	2	5 TMs	Enriched	Detected
3	PVP01_1341900	GAPM3	glideosome associated protein with multiple membrane spans 3, putative	2	6 TMs	Enriched	Detected
3	PVP01_1018200	GAP40	glideosome-associated protein 40, putative	2	9 TMs	Enriched	-
3	PVP01_0716400	GAP50	glideosome-associated protein 50, putative	2	TM, Signal	Enriched	-
3	PVP01_0505600	GAMA	GPI-anchored micronemal antigen	2	Signal, GPI	Enriched	Detected
3	PVP01_0716300	HSP70-2	heat shock protein 70, putative	2	Signal	Enriched	-
3	PVP01_0420400	HT	hexose transporter	2	12 TMs	Enriched	Detected
3	PVP01_1229700	LDH	L-lactate dehydrogenase	2	TM	Enriched	Detected
3	PVP01_0308000	PLP1	perforin-like protein 1	2	TM	Enriched	Detected
3	PVP01_1255000	RON2	rhoptry neck protein 2	2	TM, Signal	Detected	-
3	PVP01_0307900	SIAP1	sporozoite invasion-associated protein 1, putative	2	Signal	Enriched	Detected
3	PVP01_1427900	SSP3	sporozoite surface protein 3, putative	2	TM, Signal	Enriched	Detected
3	PVP01_0714500	TRX3	thioredoxin 3, putative	2	TM	-	-
3	PVP01_1132600	TLP	TRAP-like protein, putative	2	TM, Signal	-	-
3	PVP01_1339600		conserved Plasmodium protein, unknown function	1	4 TMs	Enriched	-
3	PVP01_1455800		conserved protein, unknown function	1	5 TMs, GPI	Enriched	-
3	PVP01_0710400	ICP	inhibitor of cysteine proteases, putative	1	Signal	Detected	-
3	PVP01_1025800		longevity-assurance (LAG1) protein, putative	1	6 TMs	Enriched	-
3	PVP01_0948400	MAEBL	membrane associated erythrocyte binding-like protein, putative	1	TM, Signal	-	Detected
3	PVP01_0317900	RALP1	rhoptry-associated leucine zipper-like protein 1	1	Signal	-	-
3	PVP01_0929700	SpdSyn	spermidine synthase, putative	1	TM	Enriched	-

^(a)^ Proteins were assigned priority tiers (1 is highest) based on experimental and theoretical evidence. Tier 1 = protein had predicted characteristics of a surface protein (transmembrane domain (TM), signal peptide, or glycophosphatidylinositol (GPI) anchor) and evidence for incorporation of the biotin label was observed in the identifying mass spectra. Tier 2 = spectral evidence for label only. Tier 3 = TM, signal peptide, or GPI anchor only.

^(b)^ Indicates if protein was identified in one or both of the surface labeled replicate samples.

^(c)^ Evidence used to assign tiers in (a). “Labeled” indicates evidence for incorporation of the biotin label was observed in the identifying mass spectra. Predicted characteristics of surface proteins are listed: transmembrane domain (TM), signal peptide, or glycophosphatidylinositol anchor (GPI).

^(d)^ Evidence for the protein being surface-exposed in *P*. *falciparum* salivary gland sporozoites [20]. “Enriched” indicates the protein was significantly more abundant in labeled samples versus unlabeled controls based on statistical analysis from multiple biological replicates. “Detected” indicates that the protein was detected but was not significantly enriched. “-” indicates there is an annotated *P*. *vivax* ortholog that was not detected.

^(e)^ Evidence for the protein being surface exposed in *P*. *yoelii* salivary gland sporozoites [74]. “Detected” indicates the protein was detected in either of two biological replicates. Evidence for labeling was not assessed in that experiment. “-” indicates there is an annotated *P*. *vivax* ortholog that was not detected.

## Discussion

The sole function of a *Plasmodium* sporozoite injected into the skin of the host during a mosquito bite is to find its way to the liver and initiate liver stage development. To achieve this aim, the parasite must be mobile, traverse various tissue barriers, and finally recognize and infect a hepatocyte in the liver. These complex processes rely on interaction of various parasite proteins with the host tissues and represent a bottleneck of *Plasmodium* infection, as only small fraction of sporozoites produced in a mosquito makes it to the host liver. Impediment of the parasite-host interaction presents an opportunity to interfere with the parasite life cycle.

Presented here is an effort to identify and characterize the proteins in *P*. *vivax* salivary gland sporozoites. While the total number of proteins identified with high confidence is comparable to the most comprehensive analyses of its kind performed on *P*. *falciparum* and *P*. *yoelii* sporozoites [19], the list of identified proteins presented here is not assumed to be complete. The shotgun proteomics methods for high-throughput proteomic profiling employed here are inherently biased toward highly abundant proteins and are affected by sample complexity and the dynamic range of protein concentrations. These limitations are especially pronounced when analyzing mosquito-stage *Plasmodium* parasites. Obtaining sufficient sample material for analysis is difficult, as it requires dissecting hundreds of mosquitoes and extracting the sporozoites from the salivary glands. There is unavoidable loss of sporozoites during the purification process, but this step is absolutely critical, otherwise the signal from contaminating mosquito proteins masks parasite peptides in the mass spectrometer. Assuming that there are more proteins present in sporozoites than detected here, the identification of these proteins will likely require further improvements in techniques for purifying large numbers of sporozoites along with continued improvements in mass spectrometer detection limit and duty cycle.

Previous efforts to catalogue the protein complement of *Plasmodium* sporozoites have used laboratory strains, whereas the sporozoites analyzed in this work were obtained from clinical samples isolated from natural infections. Because of the scarcity of the samples, each of the four samples analyzed here (two whole-proteome and two surface-enriched) were different field isolates of *P*. *vivax*. To account for expected polymorphism among field isolates, the mass spectra were searched against a reference proteome supplemented with protein sequences bearing polymorphisms observed in 32 different Thai field isolates. This analysis showed that 301 proteins in the samples exhibited sequence polymorphisms not found in the *P*. *vivax* P01 reference proteome. While nearly all of these of proteins could have been identified from conserved peptides present in the reference proteome, knowledge of polymorphisms gained from genomic and transcriptomic analyses of field isolates was critical for accurate analysis of the samples. At a qualitative level, the VK210 and VK247 haplotypes of CSP could be confirmed, and the VK210 whole proteome sample appeared to have contained a mixed infection of at least two different VK210 field isolates. Additionally, it was not possible to detect O-fucosylation of TRAP in some of the samples without the knowledge that the TRAP peptide containing the O-fucosylated Thr can contain an Arg→Lys substitution, a polymorphism that was present in a third of the analyzed field isolate genomes but in neither the *P*. *vivax* Sal-1 nor the *P*. *vivax* P01 reference proteomes. Protein quantification by spectral counting revealed important information about the relative abundance of proteins within and between salivary gland sporozoite samples of the same species and across species. The high-throughput proteomics methods employed here require exact knowledge of the protein sequence in order to detect component peptides, thus accurate quantification of proteins bearing amino acid substitutions required knowledge of protein sequence polymorphisms that were not reflected in the reference proteome.

Label-free protein quantification based on spectral counting was used to compare relative protein abundance within and among samples. In addition to comparing the two *P*. *vivax* salivary gland sporozoite proteomes to each other, a union list of identified *P*. *vivax* proteins was compared with a *P*. *falciparum* salivary gland sporozoite dataset obtained from re-analysis of published proteomic data [19]. The quantitative data was useful for identifying general trends, e.g., highly abundant proteins that were identified in all datasets or proteins that were abundant in one species but whose orthologs were conspicuously absent in another. Identifying a protein in one sample and not the other when comparing two similar samples is common in MS-based proteomics; a protein may not be detected because it is truly absent in the sample, because it is below the detection limit of the instrument, or due to some technical issue such as interference from some other species in the sample or the stochastic sampling of the ion stream by the mass analyzer [75]. In the comparison of the two *P*. *vivax* salivary gland sporozoite samples, the proteins identified in one sample but not the other were primarily low-abundance, suggesting that detection limit was the primary source of differences in proteome coverage between the two samples. Likewise, when the relative abundance of any given protein was compared between the two samples, high-abundance samples showed little deviation from the population mean of a 1:1 ratio, while the deviation tended to increase at lower concentrations. This increasing deviation with decreasing spectral counts is a known phenomenon in spectral counting methods [39, 44]. Statistical tests based on independent assessment of protein ratios as well as the population of protein abundances in the samples were used to identify proteins with significantly different protein abundance between samples. When biological replicates are feasible, it is common to employ a paired t-test of protein abundance ratios obtained from spectral counts [38]. When biological replicates are not available, a conservative likelihood ratio test, the G-test, can be applied to the pooled spectral counts from LC-MS technical replicates [40, 44]. The advantage of this test for spectral counting is that at low spectral counts where quantification error is high, only the largest protein ratios achieve significance. However, at high spectral counts, even protein ratios near 1:1 can be assigned significance. Based on the observation that the population of log-transformed abundance ratios of proteins detected in both samples was Gaussian, it could be assumed that only proteins at the extreme ends of the distribution were truly significant, and the complementary error function provided a *p*-value as a metric for this deviation. In conjunction with the G-test to eliminate spuriously large protein ratios obtained from low spectral counts, it was possible to identify a small number of proteins that may have had truly different protein expression between the two *P*. *vivax* samples (though technical sources of variance cannot be ruled out). Conversely, the analysis showed that the majority of identified proteins exhibited similar protein expression levels in the field isolates examined. Such observations are of value when considering targets for novel vaccines or therapeutics. The same quantitative approach used to compare the two *P*. *vivax* samples also enabled comparison of the *P*. *vivax* and *P*. *falciparum* samples and demonstrated that for most proteins identified in one species, the ortholog was identified at a similar relative abundance in the other species, especially among high-abundance proteins. In addition to proteins identified in each species for which there is no annotated ortholog in the other species, the significance thresholds provided by the statistical tests identified a number of proteins with putatively different expression in the two species that warrant further exploration, including proteins identified in one species whose ortholog was not detected at all in the other. Further investigation will be required to determine if these sorts of proteins are truly expressed at greater levels in *P*. *vivax* compared to *P*. *falciparum* and whether they may play a specialized role in *P*. *vivax* biology.

**Fig 4:**
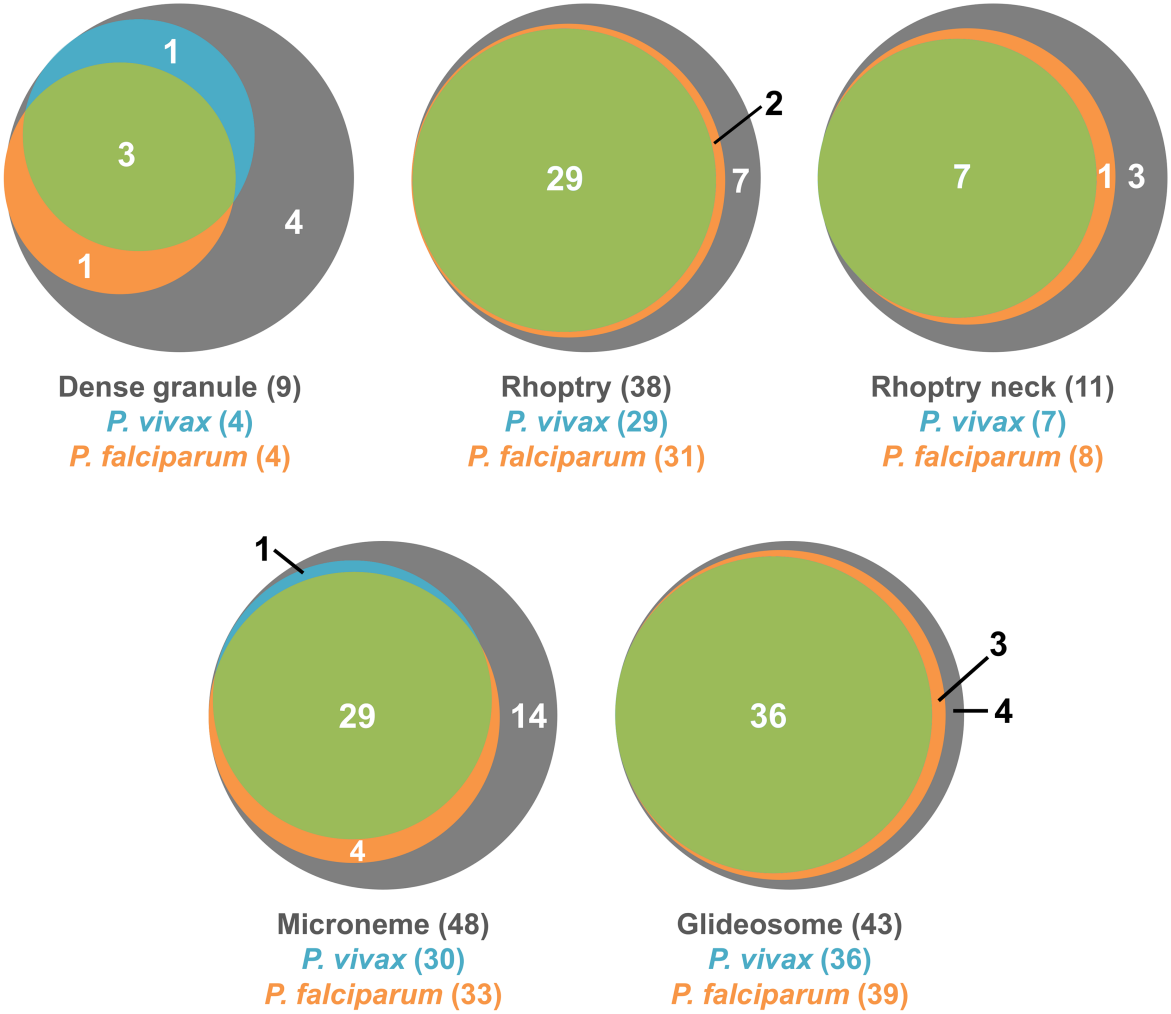
Comparison Invasion-related proteins identified in *P*. *vivax* and *P*. *falciparum* salivary gland sporozoites. Proteins identified from proteomic analyses of *P*. *vivax* and *P*. *falciparum* salivary gland sporozoites were compared against a compendium of known and putative invasion-related *Plasmodium* proteins (S10 Table). Proportional Venn diagrams show the number of proteins in each category identified only in *P*. *vivax* (cyan), only in *P*. *falciparum* (orange), in both species (green) and neither species (gray). The total number of proteins identified in each species and the total number of proteins in the category are listed below each diagram.

Examining the curated functional annotation of the *Plasmodium* proteome revealed that many of the proteins that are known or predicted to be involved in invasion in blood stages or in sporozoites of different *Plasmodium* species are also expressed in *P*. *vivax* sporozoites. Multiple annotation sources were combined in order to compile a list of dense granule, microneme, rhoptry, rhoptry neck, and glideosome proteins, and protein detection was compared between *P*. *vivax* and *P*. *falciparum* salivary gland sporozoites (Fig 4, S10 Table). There was a very high overlap in the invasion-related proteins detected in the two species, and the handful of proteins detected in only one species or the other were detected only at low abundance. For example, the most abundant of these invasion-related proteins identified in *P*. *vivax* but not *P*. *falciparum* was the micronemal protein merozoite TRAP-like protein (MTRAP; PVP01_0613800). This protein was confidently identified in both of the *P*. *vivax* samples analyzed here, though only in the second quartile of relative abundance. Its syntenic ortholog (PF3D7_1028700) was not among the 2010 proteins identified from *P*. *falciparum* sporozoites, but the transcript of MTRAP has previously been detected in *P*. *falciparum* salivary gland sporozoites [76], suggesting that the failure to detect this protein by proteomics may reflect limit of detection rather than true biological difference between the two *Plasmodium* species.

In addition to detecting the proteins present in *P*. *vivax* salivary gland sporozoites, the data were analyzed for evidence of post-translational modifications, specifically phosphorylation and glycosylation. Protein phosphorylation is of interest for development of antimalarial drugs [77, 78] because the reversible modification is involved in regulation of essentially every aspect of the complex *Plasmodium* life cycle, yet the parasite and the mammalian host are sufficiently phylogenetically divergent that many *Plasmodium* protein kinases can, in theory, be selectively inhibited [78, 79]. Although the limited amount of starting material available for this work precluded phosphopeptide enrichment, 139 proteins were identified with evidence of phosphorylation, and the orthologs of 48 of these were also phosphorylated in *P*. *falciparum* salivary gland sporozoites. The most prevalent functional class of proteins exhibiting phosphorylation was proteins involved in transcriptional and translational regulation, including DNA- and RNA-binding proteins and transcription and translation factors, suggesting active phosphorylation-mediated regulation of gene expression in this stage. Many of these proteins were also among the other prevalent class of proteins, those with ATP binding activity. Components of the gliding motility machinery were also well-represented among the observed phosphoproteins, including MyoA, the glideosome-associated proteins GAP40 and GAP45, several inner membrane complex (IMC) proteins, and the calcium-dependent protein kinase CDPK1. A phosphoproteomic analysis of *P*. *falciparum* schizonts [58] found evidence that phosphorylation helps to regulate the glideosome machinery, and *in vitro* work confirmed that GAP45, MyoA, and CDPK1, which are proteins important for motility of the merozoites that emerge from blood-stage schizonts, are substrates of protein kinase A. That these proteins are also phosphorylated in sporozoites suggests that phosphorylation also plays a role in regulating gliding motility in sporozoites. Recently, a glideosome-associated connector (GAC) protein has been identified which links the adhesin MIC2 (the *Toxoplasma gondii* ortholog of *Plasmodium* TRAP) to F-actin and is essential for motility and invasion [80]. *P*. *vivax* GAC (PVP01_1110200) was in the top decile of protein abundance in the samples analyzed here. Over half of the GAC in *P*. *vivax* sporozoites was phosphorylated at Ser10 or Ser15 on the protein N-terminus, and approximately 30% of the protein was phosphorylated at Ser1495 or Ser1496 on a section bearing homology to a protein-binding armadillo-type fold (ARM). Ser1496 on the same peptide in *P*. *falciparum* was approximately 38% phosphorylated in the salivary gland sporozoite data analyzed here. Previous phosphoproteomic analyses identified eight different phosphosites on GAC in *P*. *falciparum* blood stages [56–60], several of them on regions of conserved sequence between *P*. *falciparum* and *P*. *vivax*. The ARM phosphosite seen in *P*. *vivax* and *P*. *falciparum* sporozoites was also observed in *P*. *falciparum* blood stages, but the N-terminal phosphosites were not. AMA1 plays a role in adhesion of merozoites to erythrocytes, though its role is dispensable for rodent *Plasmodium* sporozoite invasion of hepatocytes [81]. The role of AMA1 in human-infecting *Plasmodium* sporozoites is currently undetermined. The protein has a single transmembrane domain near its C-terminus that serves as an anchor to the parasite plasma membrane. Previous proteomic analyses have shown that several residues on the cytoplasmic tail of AMA1 are phosphorylated in blood stage parasites [56–60], and mutating these residues to prevent phosphorylation resulted in a defect in invasion of erythrocytes [82]. AMA1 in *P*. *falciparum* shares an identical sequence with *P*. *vivax* at the C-terminus. In the *P*. *vivax* sporozoite samples analyzed here, the cytoplasmic tail of AMA1 was observed with a single phosphorylation at Ser551, Thr553 or Thr554 (corresponding to Ser610, Thr612 and Thr613 in *P*. *falciparum*). In the *P*. *falciparum* salivary gland sporozoite data re-analyzed here, nearby Ser588 on the cytoplasmic tail was phosphorylated. In the *P*. *falciparum* and both *P*. *vivax* sporozoite samples, the peptides containing the respective phosphosites were never observed in unmodified form, suggesting AMA1 is constitutively phosphorylated in salivary gland sporozoites. These conserved residues have been observed to be variably phosphorylated in AMA1 in *P*. *falciparum* blood stages [56, 59, 60]. These results suggest that phosphorylation of AMA1 plays a role in regulating the protein’s function in sporozoites, perhaps by mediating attachment to the glideosome.

Importantly, many of the phosphoproteins identified in the sporozoite samples have not been observed to be phosphorylated in the handful of blood-stage phosphoproteomes published to-date, including several proteins known to be specific to the sporozoite stage. Among these stage-specific phosphoproteins are proteins known to be located on the sporozoite surface. For example, it has been previously determined that sporozoite surface protein 3 (SSP3; PVP01_1427900, PF3D7_0812300) is found on the surface of *P*. *yoelii* and *P*. *falciparum* salivary gland sporozoites [19, 20, 83], and here we show that it is likely surface-exposed in *P*. *vivax* sporozoites as well. The role of SSP3 is not fully understood, but initial work suggests that it plays a role in gliding motility [83]. Approximately 30% of SSP3 in *P*. *vivax* sporozoites was phosphorylated at Ser440 near the C-terminus of the protein. A single predicted transmembrane domain at residues 402–424 is likely the point where the protein is anchored to the membrane, placing the phosphosite on the cytosolic portion of the protein. Approximately 40% of SSP3 in *P*. *falciparum* sporozoites was similarly phosphorylated at the C-terminal cytoplasmic tail at either or both of two Ser residues, Ser456 (the *P*. *falciparum* counterpart of the *P*. *vivax* S440 phosphosite) or nearby Ser459. Further experimentation will be required to elucidate any role phosphorylation might play in the function of this and other phosphorylated sporozoite surface proteins, as well any effect on their antigenicity.

It has been recently shown that the major sporozoite surface proteins CSP and TRAP are glycosylated at their TSR domains in *P*. *falciparum* salivary gland sporozoites [20] and the data presented here now show that these proteins are also glycosylated in *P*. *vivax* sporozoites. Strikingly, both CSP and TRAP in *P*. *vivax* were modified only with a single deoxyhexose (presumably O-fucose), whereas in *P*. *falciparum*, CSP was observed with either a deoxyhexose or a deoxyhexose-hexose disaccharide (likely O-fucose-β-1,3-glucose), and TRAP was observed with the O-linked mono- or disaccharide as well as with a C-linked hexose (likely C-mannose). The reason for this difference is not immediately clear. The monosaccharide-modified versions of TRAP and CSP were the dominant forms in both *P*. *falciparum* and *P*. *vivax* sporozoites, and a putative O-fucosyltransferase POFUT2 (PF3D7_0909200, PVP01_0707700), which could hypothetically add O-fucose to TSR domains, was observed to be expressed in sporozoites of both species. In *P*. *falciparum* sporozoites, the dissacharide-modified versions of CSP and TRAP were present at lower abundance than the monosaccharide-modified versions, so it is conceivable that in the *P*. *vivax* samples the disaccharide-modified versions were present but at concentrations below the detection limit. It is also possible that the necessary glycosyltransferase was not expressed. While no putative β-1,3-glucosyltransferase for adding glucose to O-fucose has been identified in *Plasmodium*, PfPIESP1 (PF3D7_0310400) has been identified as having sequence homology with human β-1,3-glucosyltransferase and possesses putative glycosyltransferase domains [20]. PIESP1 was expressed in *P*. *falciparum* salivary gland sporozoites [19], but its *P*. *vivax* homolog (PVP01_0829800) was barely detected in the *P*. *vivax* sporozoites analyzed here (identified by a only two PSMs in one sample and not at all in the other). The absence of C-linked hexose on TRAP in the *P*. *vivax* sporozoites analyzed here was unequivocal. Unlike O-linked glycans, C-mannose is not gas-phase labile and withstands collision-induced dissociation, giving rise to peptide fragment ions that precisely identify the residue to which the modification is attached. Furthermore, some portion of the C-mannose undergoes cross-ring fragmentation that gives rise to neutral loss species that further corroborate the identity of the C-mannose [20]. The glycosylated TRAP peptide was identified by dozens of spectra in the *P*. *vivax* samples, none of which contained evidence for modified Trp. A putative C-mannosyltransferase (PF3D7_0806200, PVP01_0114300) that could hypothetically add C-mannose to TSR domains was expressed in both *P*. *falciparum* [19] and *P*. *vivax* sporozoites, though this function has yet to be verified experimentally. Interestingly, this disparity in glycosylation was also observed in TRAP expressed in mammalian cells: *Pf*TRAP was C-mannosylated but *Pv*TRAP was not [55]. It is notable that in *Pv*TRAP, the sequence where it would be expected to find C-mannosylation contains prolines that could affect the secondary structure of the motif and disrupt recognition by the glycosyltransferase. Further studies will be required to determine whether the observed differences in CSP and TRAP glycosylation between *P*. *falciparum* and *P*. *vivax* are due to differences in enzyme function or some other technical or biological reason. Importantly for design of vaccine antigens, O-fucosylation of CSP and TRAP almost certainly affect antigenicity of the proteins. Structural studies of these proteins have shown that the glycans project above the protein surface and yet have structurally constrained orientations [54, 55, 84], and studies of the conserved TSR domain in other species have shown that fucosylated amino acids may be viewed as surrogate amino acids [85], so the protein and carbohydrate elements create unique combinatorial epitopes.

A chemical labeling approach was used to enrich proteins that are surface-exposed on salivary gland sporozoites. As previously discussed [20, 73], this surface biotinylation approach is known to produce spurious results due to labeling of cytosolic proteins presumably arising from a portion of sporozoites that exhibit compromised plasma membranes, an inevitable byproduct of the excessive sample handling involved in dissecting, purifying, and labeling the parasites. The data presented here were curated with theoretical and experimental information to select those identified proteins that are the most likely to be truly surface-exposed and proteins were assigned priority tiers based on this evidence in order to identify high-quality candidates for future efforts to validate and test these proteins as vaccine antigens. This approach is supported by the fact that the list of high-quality candidates includes several known sporozoite surface proteins, including CSP, TRAP, SSP3, and SPELD. Additionally, cross-referencing the results with similar analyses of *P*. *falciparum* [20] and *P*. *yoelii* [74] salivary gland sporozoites revealed a large overlap in the proteins that the technique identified across species. A notable exception was a 6-Cys protein (PVP01_0303900) that has no ortholog in *P*. *falciparum* or *P*. *yoelii* but does have syntenic orthologs in the more closely related malaria parasites *P*. *knowlesi* and *P*. *cynomolgi*. In other *Plasmodium* species, other 6-cys proteins are known to be found on the sporozoite surface and to play a role in liver invasion [20, 86]. Future studies will determine what role this putative surface protein may have in sporozoites and if it will be useful as a vivax-specific antigen.

The putatively surface-exposed proteins identified here as well as in *P*. *falciparum* and *P*. *yoelii* sporozoites included known cytosolic proteins. While there remains the possibility that these results represent experimental artifact as discussed, there is increasing evidence that cytosolic proteins can have “moonlighting” roles and be found on the cell surface of *Plasmodium* and other organisms. For example, the chaperone HSP70-2 (BiP) has been shown to localize at the surface of certain cell types in other organisms [87], and HSP70-2/BiP (PVP01_0716300, PF3D7_0917900) was identified as putatively surface-exposed in both *P*. *vivax* and *P*. *falciparum* salivary gland sporozoites. Another protein classified as a heat shock protein, HSP20, has been identified by biotinylation of sporozoite surface proteins in both *P*. *falciparum* and *P*. *vivax* sporozoites. This protein has been demonstrated to be surface-exposed in *P*. *berghei* salivary gland sporozoites by immunoelectron microscopy [88]. Other intracellular proteins repeatedly identified as surface-exposed by the surface labeling technique include components of the gliding motility machinery, including actin, MyoA, glideosome-associated proteins (GAP), and inner membrane complex (IMC) proteins. Immunofluorescence assays of un-permeabilized *P*. *falciparum* salivary gland sporozoites showed that the glideosome proteins GAP45 and MTIP were accessible to antibodies during gliding [20], though whether this was due to relocation of the proteins to the sporozoite surface or permeability of the plasma membrane in gliding sporozoites is not known. Similarly, immunofluorescence identified actin, which is part of the gliding machinery, at the ookinete surface [71]. Another glideosome-associated protein, GAP50, has been shown to relocate to the surface of gametes where it binds complement regulator proteins and inactivates human complement in the blood meal that would otherwise induce lysis of the parasite [89]. Taken together, this information suggests that even “known” intracellular proteins identified by this surface labeling method can reflect truly surface-exposed proteins and warrant further investigation.

In conclusion, the MS-based proteomics methods employed here enabled the most comprehensive identification to-date of proteins and their post-translational modifications present in *P*. *vivax* sporozoites. Combined with the identification of putatively surface-exposed proteins of *P*. *vivax* salivary gland sporozoites, these results suggest that the complement of surface-exposed proteins on salivary gland sporozoites may contain many unexpected as well as post-translationally modified proteins that warrant further experimentation to verify their localization and assess their suitability as vaccine antigens.

## Supporting information

S1 File Extended methods. (DOCX)

S2 File *P*. *vivax* Sal-1 proteins with variant sequences. All *P*. *vivax* proteins identified from DNA-seq and RNA-seq analyses of Thai field isolates whose protein sequences differ from the *P*. *vivax* Sal-1 reference proteome. File is in protein fasta format.
(ZIP)

S1 Fig Distributions of NSAF values of identified *P*. *vivax* proteins. (PDF)

S2 Fig Distributions of NSAF values of identified *P*. *vivax* and *P*. *falciparum* proteins. (PDF)

S3 Fig Mass spectral evidence for circumsporozoite protein (CSP) haplotype. (PDF)

S4 Fig Mass spectrometry reveals sequence polymorphisms in thrombospondin-related anonymous protein (TRAP). (PDF)

S5 Fig Evidence for glycosylation of CSP in *P*. *vivax* VK210 salivary gland sporozoites. (PDF)

S6 Fig Evidence for glycosylation of CSP in *P*. *vivax* VK247 salivary gland sporozoites. (PDF)

S7 Fig Evidence for glycosylation of TRAP in *P*. *vivax* VK210 salivary gland sporozoites. (PDF)

S8 Fig Evidence for glycosylation of TRAP in *P*. *vivax* VK247 salivary gland sporozoites. (PDF)

S1 Table Extended LC-MS methods parameters. (XLSX)

S2 Table Proteins identified from whole proteome analysis of *P*. *vivax* salivary gland sporozoites. (XLSX)

S3 Table Comparison of proteins identified from whole proteome analysis of *P*. *falciparum* and *P*. *vivax* salivary gland sporozoites. (XLSX)

S4 Table Frequency of protein sequence polymorphisms in Thai *P*. *vivax* strains. (XLSX)

S5 Table Protein sequence variants detected in *P*. *vivax* salivary gland sporozoites. (XLSX)

S6 Table Phosphoproteins identified in *P*. *vivax* and *P*. *falciparum* salivary gland sporozoites. (XLSX)

S7 Table Phosphorylated peptides identified from mass spectrometric analysis of *P*. *vivax* salivary gland sporozoites. (XLSX)

S8 Table Phosphorylated peptides identified from mass spectrometric analysis of *P*. *falciparum* salivary gland sporozoites. (XLSX)

S9 Table All proteins identified from surface labeling of live *P*. *vivax* salivary gland sporozoites with biotin. (XLSX)

S10 Table Identification of invasion proteins in salivary gland sporozoites. (XLSX)
