# Supplementary material for: Proteogenomic analysis of the total and surface-exposed proteomes of Plasmodium vivax salivary gland sporozoites
Source: PLoS Negl Trop Dis. 2017 Jul 31;11(7):e0005791. doi: 10.1371/journal.pntd.0005791 (PMC5552340; doi:10.1371/journal.pntd.0005791)
Supplement: S1 File — (DOCX) [file pntd.0005791.s001.docx]

**S1 FILE. EXTENDED METHODS**

**S1a. Mass spectrometry data analysis**

**S1b. Protein quantification by spectral counting**

**S1c. Extended details of 1D SDS-PAGE fractionation and in-gel tryptic digestion**

**S1d. References**

**S1a. Mass spectrometry data analysis**

The MS data generated for this manuscript, along with the search parameters, analysis parameters and protein databases can be downloaded from PeptideAtlas (www.peptideatlas.org) using the identifiers PASS00976 (whole proteome) and PASS00977 (surface-labeled). Mass spectrometer output files were converted to mZML format using msConvert version 2.2.0 (whole proteome data) or 3.0.5533 (surface-labeled data) [1] and searched with Comet version 2015.02 rev.0 [2].

The default value was used for most Comet paramters. Salient Comet parameters were as follows:

peptide_mass_tolerance = 10 ppm

mass_type_parent = monoisotopic masses

mass_type_fragment = monoisotopic masses

precursor_tolerance_type = MH+ (default)

isotope_error = off

search_enzyme_number = trypsin

num_enzyme_termini = 1

allowed_missed_cleavage = 2

*variable modifications format:
 <mass> <residues> <0=variable/1=binary> <max_mods_per_peptide> <term_distance> <n/c-term> <required>*

variable_mod01 = 15.994915 M 0 3 -1 0 0

O*xidation of Met*

variable_mod02 = 42.010565 n 0 1 1 0 0

*Acetylation of protein N-terminus at N-terminal residue or next residue*

variable_mod03 = -17.026549 QC 0 1 0 2 0

*Formation of pyro-Glu from N-terminal Gln; formation of S-*

*carbamoylmethylcysteine from deamidation by cyclization of N-terminal*

*carbamidomethylated Cys*

variable_mod04 = -18.010565 E 0 1 0 2 0

*Formation of pyro-Glu from N-terminal Glu*

variable_mod05 = 79.966331 STY 0 3 -1 0 0

*Phosphorylation. Only used for phosphorylation search.*

max_variable_mods_in_peptide = 5

fragment_bin_tol = 1.0005

fragment_bin_offset = 0.4

theoretical_fragment_ions = M peak only

max_fragment_charge = 3

max_precursor_charge = 6

The MS/MS data were analyzed using the Trans-Proteomic Pipeline (TPP) [3] version 5.0.0 Typhoon. Peptide spectrum matches (PSM) were assigned scores in PeptideProphet [4], peptide-level scores were assigned in iProphet [5], and Protein identifications were inferred with ProteinProphet [6].

The TPP was run in the command line as follows:

>InteractParser FILENAME.interact.pep.xml INPUTFILES.pep.xml –I

-I writes precursor intensity to the .pep.xml file

>RefreshParser FILENAME.interact.pep.xml database.fasta

>PeptideProphetParser FILENAME.interact.pep.xml ACCMASS PPM MINPROB=0 DECOY=DECOY NONPARAM EXPECTSCORE

ACCMASS Employs the accurate mass model to correct for systematic

mass shift.

PPM Enables use of PPM mass tolerance in ACCMASS

MINPROB=0 All spectra are used to build the mixture models

(default MINPROB=0.05)

DECOY=DECOY Gene ID’s starting with DECOY are decoy proteins

NONPARAM Use non-parametric mixture models

EXPECTSCORE Use the Comet expect score as the primary discriminant

for building mixture models

DECOYPROBS Decoy proteins are assigned PeptideProphet scores and

kept in the file. This flag was only used when manually

calculating a decoy-estimated FDR.

>RefreshParser FILENAME.interact.pep.xml database.fasta

>InterProphetParser NONSP FILENAME.interact.pep.xml FILENAME.interact.iproph.pep.xml

NONSP Do not use the “number of sibling peptides” model. This model is

applied in ProteinProphet, so it is unnecessary/inappropriate here.

>ProteinProphet FILENAME.interact.iproph.pep.xml FILENAME.interact.iproph.prot.xml IPROPHET NORMPROTLEN

IPROPHET Input data are from iProphet.

NORMPROTLEN Normalize score to protein length

When searching the data for evidence of phosphorylation, the DECOYPROBS flag was used in PeptideProphet so that decoy PSM matching decoy protein entries were assigned probabilities and included in the final output. The one percent FDR for all PSM and for PSM matching phosphorylated peptides was found by selecting the PeptideProphet probability at which 1% of the PSM matched decoy proteins. If a PSM matched a decoy protein and a real protein, it was not considered a decoy match.

Localization of phosphate groups within phosphopeptides was confirmed and/or corrected using a development version of PTMProphet (source code available at https://sourceforge.net/p/sashimi, SVN revision number 7584. The listed variable modifications and neutral losses were considered by the program when assessing the best localization for all observed variable modifications.

>PTMProphetParser STY:79.966:-97.97690,MW:15.9949,E:-18.010565,Q:-17.026549,C:-17.026549,n:42.010565 MZTOL=0.2 FILENAME.interact.pep.xml

STY:79.966:-97.97690 Phosphorylation of Ser, Thr, or Tyr. Also uses neutral loss of H_3_PO_4_

MW:15.9949 Oxidation of Met or Trp

E:-18.010565 Deamidation from formation of pyro-Glu at N-terminal Glu

Q:-17.026549 Deamidation from formation of pyro-Glu at N-terminal Gln

C:-17.026549 Deamidation at N-terminal carbamidomethylated Cys for from formation of S-carbamoylmethylcysteine

n:42.010565 N-terminal acetylation

MZTOL=0.2 Fragment mass tolerance of ±0.2 m/z

**S1b. Protein quantification by spectral counting**

Relative protein abundance within and between samples was estimated using a label-free proteomics method based on spectral counting. The spectral counts for a given protein was taken as the number of peptide spectrum matches (PSMs) identifying the protein in the PeptideProphet results. PeptideProphet probabilities are assigned at the spectrum level (as opposed to the peptide level in iProphet and the protein level in ProteinProphet). Both iProphet and ProteinProphet use PSMs that score poorly at the spectrum level to build their models, so while the peptide probabilities in iProphet and the protein probabilities in ProteinProphet are robust, the counts of PSM associated with a give proteins obtained from either of those programs include low-quality spectra. For this reason, we chose to obtain spectral counts directly from PeptideProphet output. For each sample, the mass spectra from all combined gel fractions and LC-MS/MS technical replicates were pooled and analyzed together in the TPP. Decoy entries were used to build the mixture models, but decoy entries were not assigned PeptideProphet probabilities. PSMs were taken for spectral counting if they were identified at a PeptideProphet probability corresponding to a false discovery rate (FDR) less than 1.0 % according to the PeptideProphet models.

PSMs from degenerate peptides (peptides whose sequences are found in multiple proteins in the database) were split among proteins containing that peptide in a weighted fashion similar to the approach described in the Abacus program [7, 8], but executed manually in Microsoft Excel. First, multi-protein entries were identified. Multi-protein entries are cases when a single protein identification in ProteinProphet contains multiple gene IDs, separated by commas, because the detected peptides map to multiple proteins that cannot be distinguished based on the available data. A single gene ID (the first to appear in alphabetical order) was selected as the entry identifier. All multi-protein entries were identified, including those assigned a ProteinProphet probability of zero. In the PeptideProphet results, the multiple gene IDs associated with multi-protein entries were replaced with the single entry identifier gene ID. Then all PSM were separated into degenerate peptides (those still mapping to multiple gene IDs after collapsing the multi-protein entries from ProteinProphet) and the non-degenerate peptides. Each identified peptide was reduced to its stripped sequence, i.e., its amino acid sequence disregarding variable modifications and charge state. For each degenerate peptide, the following operations were performed:

1. Identify all proteins to which the peptide maps.
2. For each of the proteins identified in step 1, determine the total number of PSMs identifying non-degenerate peptides mapping to that protein.
3. Divide the degenerate peptide’s PSMs among the multiple proteins to which it maps proportional to the fraction of each protein’s non-degenerate PSMs compared to the sum of all the non-degenerate PSMs of all the proteins to which the peptide maps. That is, if a degenerate Peptide X has X PSMs and maps to Protein A that has A non-degenerate PSMs, and Peptide X also maps to Protein B that has B non-degenerate PSMs, then Protein A is assigned X*(A/[A+B]) PSMs and Protein B is assigned X*(B/[A+B]) PSMs. If Protein A has non-degenerate PSMs and Protein B does not, Protein A receives all X PSMs. If neither protein has non-degenerate PSMs, then the PSMs from Peptide X are not assigned to any protein.
4. For each protein, find the sum of all PSMs from all peptides, including non-degenerate PSMs and degenerate PSMs that were divided in a weighted fashion. This value will be reported as the total PSMs and will be used for spectral counting.

Using the above approach, all proteins used for spectral counting must be identified from at least one non-degenerate peptide. In practice, occasionally proteins that passed the one percent FDR cut-off at the ProteinProphet level will not be assigned any PSM. For the five *P. vivax* data sets analyzed here, this only affected a total of five proteins that were identified from a single peptide and only one or two PSMs. The analysis of the *P. falciparum* data set was complicated by the fact that many gene ID’s are associated with up to three distinct transcript ID’s which may have slightly different amino acid sequences. In most cases the identified peptides did not distinguish among the multiple transcript IDs and all transcripts could be collapsed to a single gene ID. However, this had to be investigated on a case-by-case basis based on the ProteinProphet results.

Relative protein abundance within samples was ranked using the normalized spectral abundance factor [9, 10]. This information was used to calculate the relative abundance percentile for each sample as well as the comparison of relative abundance in Figure 1A and Figure 2A. The spectral abundance factor (SAF) for a given protein was calculated as the quotient of the PSMs (calculated as described above) and the protein's length. This normalization accounts for the fact that a longer protein would be expected to produce more tryptic peptides and thus more PSMs at the same concentration. The SAF for each *Plasmodium* protein was normalized to the sum of all *Plasmodium* SAF values obtained from the same sample to produce the normalized SAF (NSAF). This normalization accounts for different sample complexity between samples due to different amounts of sample loaded, different levels of fractionation, etc. NSAF values were natural log-transformed to ln(NSAF) [9, 10]. The population of ln(NSAF) values for each sample assumed a normal distribution (S1 Fig, S2 Fig). The natural log of the protein abundance fold-change ratio between samples was calculated as ln(NSAF)_A_-ln(NSAF)_B_ where A and B are two different samples in which the same protein is observed. (Ratios could not be produced for proteins detected in one sample but not the other). Each of these distributions was fit with a Gaussian curve in Microsoft Excel using minimum residual sum of squares and goodness of fit was evaluated with the R^2^ coefficient of determination. The standard deviation of this fit was used to estimate the variance with respect to protein abundance in Figure 1A and Figure 2A. In order to produce the plot of *P. vivax* vs *P. falciparum*, the ln(NSAF) values from the two *P. vivax* samples were combined by taking the average ln(NSAF) value for proteins detected in both samples and by taking the available ln(NSAF) value for proteins detected in only one sample.

When assessing the relative abundance of proteins between the two samples, it was desirable to estimate the relative abundance of proteins detected in one sample but not the other, which requires a non-zero value of spectral counts for the undetected protein. When quantifying proteins by spectral counts, the detection limit is effectively one spectral count. Increasing all PSM by 1 assigns undetected proteins with the lowest possible number of PSMs [11]. This adjustment skews protein ratios more heavily at low spectral counts than high spectral counts, e.g. 5:1 = 5.0 and (5+1):(1+1) = 3.0 whereas 500:100 = 5.00 and (500+1):(100+1) = 4.96. However, as will be discussed below, ratios derived from low numbers of spectral counts are known to have high errors and were addressed accordingly. In order to normalize for variable sample amount and complexity, the adjusted spectral counts (PSM+1) were normalized so that the sum of all PSM was the same in both samples as

$$c_{A}={(PSM+1)}_{A}\left[ \frac{\sum\left( PSM+1 \right)_{B}}{\sum\left( PSM+1 \right)_{A}} \right]$$

where $c_{A}$ is the adjusted and normalized spectral counts for a protein in sample A, ${(PSM+1)}_{A}$ is the adjusted number of PSM for that protein in that sample, $\sum\left( PSM+1 \right)_{A}$ is the sum of all adjusted PSM values ($PSM+1)$ for all proteins in sample A, and $\sum\left( PSM+1 \right)_{B}$ is the sum of all adjusted PSM values for all proteins in sample B. In the comparison of *P. vivax* samples, the VK247 sample, which had a higher total number of PSM, was adjusted so that the sum of the adjusted spectral counts matched the sum of of adjusted spectral counts in the VK210 sample. When comparing the *P. vivax* samples to the *P. falciparum* salivary gland sporozoite proteome, the PSM from the two *P. vivax* samples and the *P. falciparum* sample were first adjusted as above to obtain non-zero values for all protein orthologs identified in one species but not the other. Proteins with no orthologs were excluded from this analysis. The spectral counts for the *P. vivax* VK247 and the *P. falciparum* sample were normalized as above to match the *P. vivax* VK210 sample, which had fewer total PSM than either of the other two samples. The two *P. vivax* samples were then combined by taking the average of the adjusted and normalized spectral counts for each protein.

The abundance ratio for a given protein between a two samples was calculated as

$$R_{A:B}=\frac{c_{A}}{c_{B}}$$

where R_A:B_ is the protein abundance ratio of a protein between sample A and sample B and c_A_ and c_B_ are the adjusted and normalized spectral counts for the protein in sample A and sample B, respectively. In order to assess the error in spuriously high protein ratios obtained from proteins with low spectral counts, the G-test of significance was applied to the corrected and adjusted PSM for each protein pair as

$$G=2\left[ c_{A}ln\left( \frac{c_{A}}{\left( \frac{c_{A}+c_{B}}{2} \right)} \right)+c_{B}ln\left( \frac{c_{B}}{\left( \frac{c_{A}+c_{B}}{2} \right)} \right) \right]$$

and a *p*-value was assigned by calculating the probability that a $\chi^{2}$ distribution with one degree of freedom was more extreme than the G statistic [11]. The $\chi^{2}$ test was performed for each G-test value in Microsoft Excel using the function =CHISQ.DIST.RT(<G statistic>,1). In order to identify protein ratios that were significantly more extreme than the normal distribution of protein ratios in the sample, all protein ratios were log-transformed by taking ${log}_{2}(R_{A:B}$). The distribution of the these values for all proteins detected in both samples was fit with a Gaussian curve using minimum residual sum of squares and goodness of fit was evaluated with the R^2^ coefficient of determination (S1 Fig, S2 Fig). Protein abundance ratios were corrected for systematic bias by subtracting the mean of this distribution (which was near 0 in all cases) from each log-transformed protein ratio. In order to assess the likelihood that a protein ratio was more extreme than the normal distribution of protein ratios, a *p-*value was calculated for each ratio using the complementary error function as

$$p=ERFC\left| \frac{{{log}_{2}(R}_{A:B})-\mu}{\sigma\sqrt{2}} \right|$$

where µ is the mean and σ is the standard deviation of the fit Gaussian. The FDR arising from multiple hypothesis testing was assessed by the Benjamini-Hochberg method for both tests independently, and proteins ratios with an FDR less than 5% by both the G-test and ERFC were considered significant.

**S1c. Extended details of 1D SDS-PAGE fractionation and in-gel tryptic digestion**

SDS-PAGE prefractionation and in-gel tryptic digestion were performed essentially as described in [12]. Samples were electrophoresed through a 4-20 % w/v SDS-polyacrylamide gel (Pierce Precise Tris-HEPES). Gels were stained with Imperial Stain (Thermo Fisher Scientific) and destained in Milli-Q Water (Millipore). The VK210 whole proteome gel was run out 52 mm and cut into 27 fractions using a grid cutter (Gel Company). The extracted peptides were pooled from fractions one and two, three and four, 22 and 23, 24 and 25, and 26 and 27 to produce a total of 22 samples. The VK247 whole proteome gel was run out 58 mm and cut into 29 fractions. The extracted peptides were pooled into a total of 15 fractions by combining fractions one and two, three and four, etc. The three surface-labeled samples (VK210 labeled, VK247 labeled, and VK247 unlabeled) were run on the same gel. Each lane was cut free-hand into eight fractions, and eight fractions for each sample were analyzed by LC-MS. Gel fractions were cut into pieces ~1 mm on a side and placed in 96-well plates and covered with a coverslip (Greiner Bio-One part number 676070). The bottom of each well was pierced twice with a 28 gauge lancet. The holes were sufficiently small that buffer did not leak out of the well, but buffer could easily be completely removed from the wells in bulk either by vacuum manifold or by centrifuge. A plastic bead placed in each well prevented the gel pieces from occluding the holes. The two whole-proteome samples were processed by in-gel tryptic digestion using a TECAN Freedom Evo. Unless otherwise stated, all incubations were performed on a thermomixer at 36 °C while vortexing at 700 RPM. Gel pieces were de-stained three times with 50 µL of 50 mM ammonium bicarbonate (ABC) in 50% acetonitrile (ACN) to each well and incubating 10 min. Gel pieces were dehydrated two times with 50 µL of ACN for 5 min. Residual ACN was removed by drying at 45 °C. Disulfide bonds were reduced by adding 50 µL of 10 mM dithiothreitol (DTT) in 100 mM ABC and incubating 30 min. The DTT solution was removed and cysteines were alkylated by adding 50 µL of 50 mM iodoacetamide in 100 mM ABC and incubating 20 min. Gel pieces were washed three times with ABC in 50% ACN and dehydrated three times with ACN as above. 50 µL of 6.25 ng/mL trypsin (Promega sequencing grade) was added to each well and incubated 4.5 hours. The supernatant was recovered and peptides were extracted by incubating the gel pieces 30 min with 40 µL of 2% v/v ACN/1% v/v formic acid. Supernatant was recovered and a second extraction was performed for 30 min with 40 µL of ACN. Supernatant was recovered and a third extraction was performed with 40 µL of 2% v/v ACN/1% v/v formic acid. The three extractions were combined with the digest supernatant, evaporated to dryness in a rotary vacuum, and reconstituted in 20 µL of HPLC loading buffer consisting of 2% v/v ACN/0.2% v/v trifluoroacetic acid. The surface-labeled gel fractions were manually processed in a fashion similar to that used for whole-proteome samples, with the following differences: gel pieces were de-stained twice for 10 min and dehydrated once for 10 min; the reduction and alkylation steps were performed with 75 µL of reagent; between the reduction and alkylation steps, the gel pieces were dehydrated by incubating 10 min with ACN at room temperature without shaking; after alkylation the gel pieces were dehydrated by incubating in 150 µL of ACN for 30 min at room temperature without shaking; gel pieces were incubated 2 h at 4 °C after adding trypsin to allow trypsin to permeate gel, and additional ABC buffer was added to ensure that all gel pieces were covered [13]; the trypsin digestion took place for 16 h in an oven at 37 °C; and peptides were extracted with consecutive 30-min washes of 40 µL of 2% v/v ACN/1% v/v formic acid, 60 % v/v ACN/0.5 % formic acid, and 100% ACN.

**S1d. References**

1. Kessner D, Chambers M, Burke R, Agus D, Mallick P. ProteoWizard: open source software for rapid proteomics tools development. Bioinformatics. 2008;24(21):2534-6. doi: 10.1093/bioinformatics/btn323. PubMed PMID: 18606607; PubMed Central PMCID: PMC2732273.

2. Eng JK, Jahan TA, Hoopmann MR. Comet: an open-source MS/MS sequence database search tool. Proteomics. 2013;13(1):22-4. Epub 2012/11/14. doi: 10.1002/pmic.201200439. PubMed PMID: 23148064.

3. Deutsch EW, Mendoza L, Shteynberg D, Slagel J, Sun Z, Moritz RL. Trans-Proteomic Pipeline, a standardized data processing pipeline for large-scale reproducible proteomics informatics. Proteomics Clin Appl. 2015. Epub 2015/01/30. doi: 10.1002/prca.201400164. PubMed PMID: 25631240.

4. Keller A, Nesvizhskii AI, Kolker E, Aebersold R. Empirical statistical model to estimate the accuracy of peptide identifications made by MS/MS and database search. Anal Chem. 2002;74(20):5383-92. Epub 2002/10/31. PubMed PMID: 12403597.

5. Shteynberg D, Nesvizhskii AI, Moritz RL, Deutsch EW. Combining results of multiple search engines in proteomics. Mol Cell Proteomics. 2013;12(9):2383-93. doi: 10.1074/mcp.R113.027797. PubMed PMID: 23720762; PubMed Central PMCID: PMCPMC3769318.

6. Nesvizhskii AI, Keller A, Kolker E, Aebersold R. A statistical model for identifying proteins by tandem mass spectrometry. Anal Chem. 2003;75(17):4646-58. Epub 2003/11/25. PubMed PMID: 14632076.

7. Fermin D, Basrur V, Yocum AK, Nesvizhskii AI. Abacus: a computational tool for extracting and pre-processing spectral count data for label-free quantitative proteomic analysis. Proteomics. 2011;11(7):1340-5. doi: 10.1002/pmic.201000650. PubMed PMID: 21360675; PubMed Central PMCID: PMCPMC3113614.

8. Zhang Y, Wen Z, Washburn MP, Florens L. Refinements to label free proteome quantitation: how to deal with peptides shared by multiple proteins. Anal Chem. 2010;82(6):2272-81. doi: 10.1021/ac9023999. PubMed PMID: 20166708.

9. Zybailov B, Mosley AL, Sardiu ME, Coleman MK, Florens L, Washburn MP. Statistical analysis of membrane proteome expression changes in Saccharomyces cerevisiae. J Proteome Res. 2006;5(9):2339-47. Epub 2006/09/02. doi: 10.1021/pr060161n. PubMed PMID: 16944946.

10. Gokce E, Shuford CM, Franck WL, Dean RA, Muddiman DC. Evaluation of normalization methods on GeLC-MS/MS label-free spectral counting data to correct for variation during proteomic workflows. J Am Soc Mass Spectrom. 2011;22(12):2199-208. Epub 2011/09/29. doi: 10.1007/s13361-011-0237-2. PubMed PMID: 21952779.

11. Hendrickson EL, Xia Q, Wang T, Leigh JA, Hackett M. Comparison of spectral counting and metabolic stable isotope labeling for use with quantitative microbial proteomics. Analyst. 2006;131(12):1335-41. Epub 2006/11/25. doi: 10.1039/b610957h. PubMed PMID: 17124542; PubMed Central PMCID: PMC2660848.

12. Lindner SE, Swearingen KE, Harupa A, Vaughan AM, Sinnis P, Moritz RL, et al. Total and putative surface proteomics of malaria parasite salivary gland sporozoites. Mol Cell Proteomics. 2013;12(5):1127-43. doi: 10.1074/mcp.M112.024505. PubMed PMID: 23325771; PubMed Central PMCID: PMCPMC3650326.

13. Shevchenko A, Tomas H, Havlis J, Olsen JV, Mann M. In-gel digestion for mass spectrometric characterization of proteins and proteomes. Nat Protoc. 2006;1(6):2856-60. doi: 10.1038/nprot.2006.468. PubMed PMID: 17406544.
