# Supplementary material for: Proteogenomic analysis of the total and surface-exposed proteomes of Plasmodium vivax salivary gland sporozoites
Source: PLoS Negl Trop Dis. 2017 Jul 31;11(7):e0005791. doi: 10.1371/journal.pntd.0005791 (PMC5552340; doi:10.1371/journal.pntd.0005791)
Supplement: S1 Fig — (PDF) [file pntd.0005791.s003.pdf]

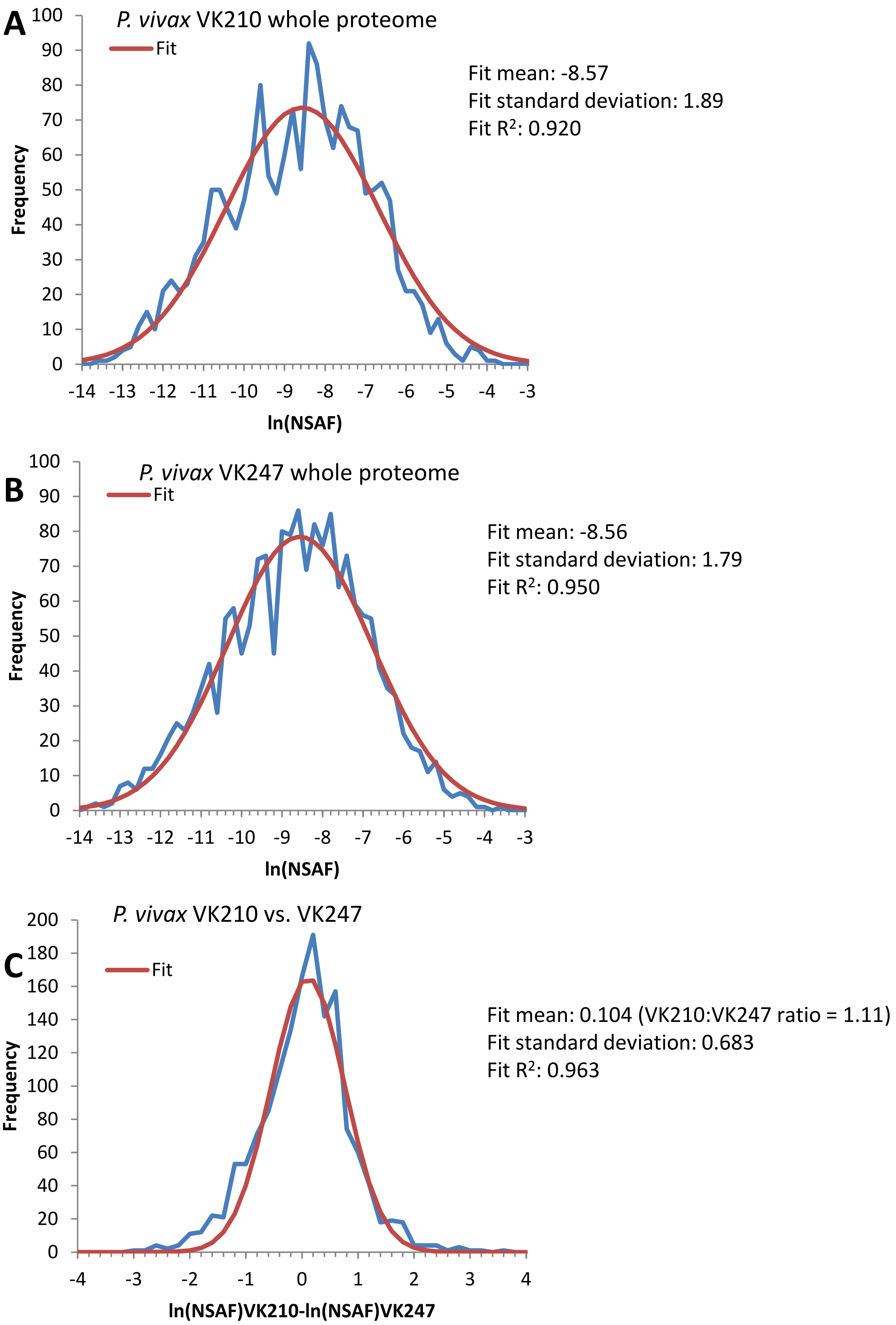

S1 Fig. Distributions of NSAF values of identified *P. vivax* proteins. Protein abundances based on spectral counts were estimated using the normalized spectral abundance factor (NSAF). The populations of natural log-transformed NSAF values of (A) the *P. vivax* VK210 salivary gland sporozoite whole proteome analysis and (B) the *P. vivax* VK247 salivary gland sporozoite whole proteome sample exhibited normal distributions with similar means and standard deviation. For each protein detected in both samples, the natural log of the protein ratio of the NSAF values observed in the VK210 sample and the VK247 sample was calculated as $\ln[NASF]_{VK210} - \ln[NSAF]_{VK247}$. The population of these values (C) produced a Gaussian distribution centered near zero, corresponding to a mean ratio of 1:1. The fit curve (red) for each population was produced by finding the minimum residual sum of squares of a curve with the equation $f(x)=A*\exp[-(x-\mu)^2/2\sigma^2]$ where A is the maximum, μ is the mean, and σ is the standard deviation. The goodness of fit was estimated by an R^2 coefficient of determination between the observed and fit values. Note that in (C) the largest residuals from the fit were from proteins with a higher observed abundance in the VK247 sample versus the VK210 sample. More proteins and peptide spectrum matches were observed in the VK247 sample, suggesting that these differences were due to limit of detection.
