# Supplementary material for: Proteogenomic analysis of the total and surface-exposed proteomes of Plasmodium vivax salivary gland sporozoites
Source: PLoS Negl Trop Dis. 2017 Jul 31;11(7):e0005791. doi: 10.1371/journal.pntd.0005791 (PMC5552340; doi:10.1371/journal.pntd.0005791)
Supplement: S2 Fig — (PDF) [file pntd.0005791.s004.pdf]

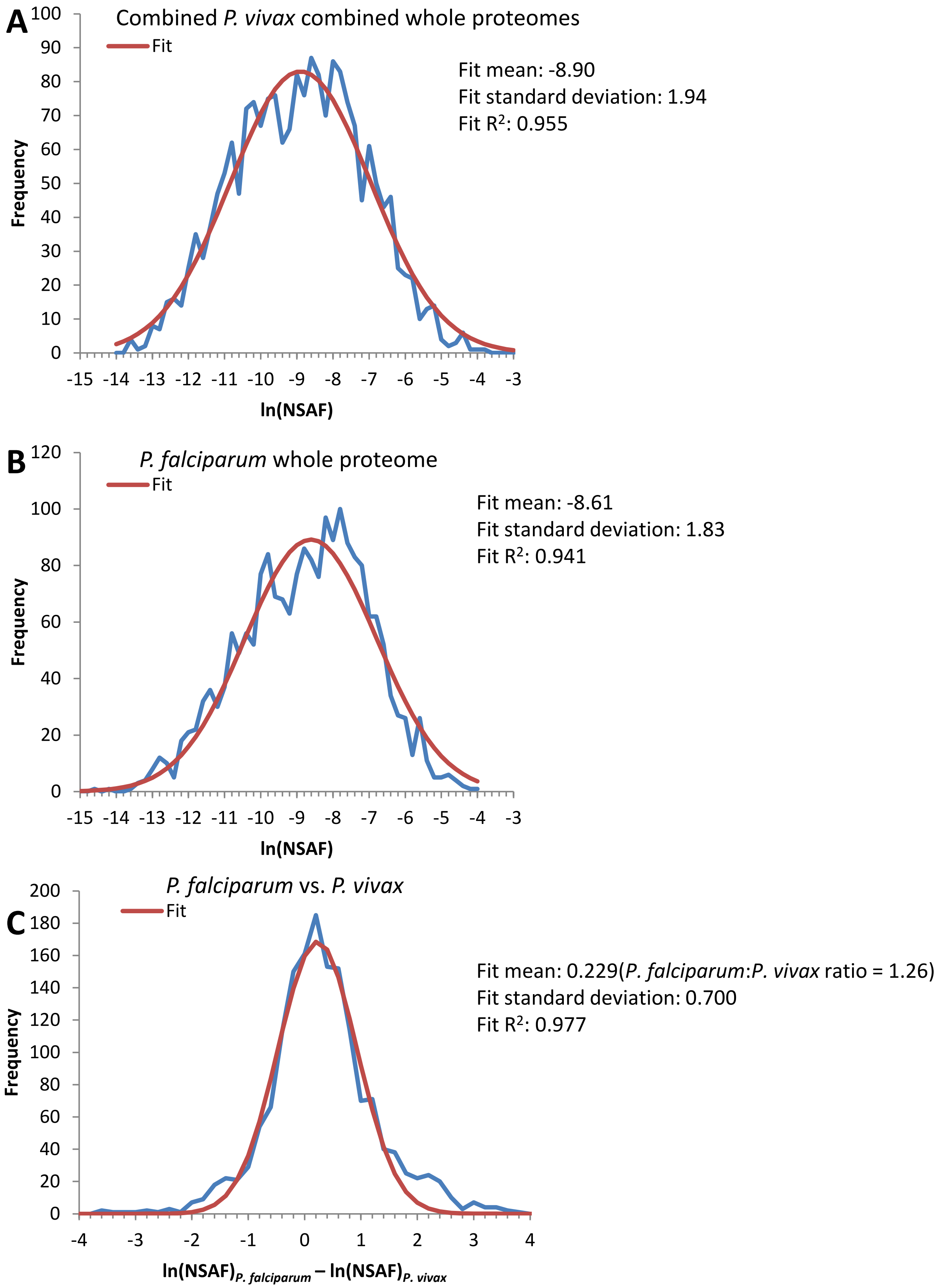

S2 Fig. Distributions of NSAF values of identified *P. vivax* and *P. falciparum* proteins. Protein abundances based on spectral counts were estimated using the normalized spectral abundance factor (NSAF). The distributions of (A) the average ln(NSAF) values of the combined *P. vivax* salivary gland sporozoite whole proteome analyses and (B) the ln(NSAF) of the re-analysis of the *P. falciaprum* salivary gland sporozoite whole proteome. Both populations exhibited normal distributions with similar means and standard deviations. For each protein ortholog detected in both samples, the natural log of the protein ratio of the NSAF values observed in both species was calculated as $\ln(\mathrm{NASF})_{Pf} - \ln(\mathrm{NSAF})_{Pv}$. The population of these values (C) produced a normal distribution centered near zero, corresponding to a mean ratio of 1:1. The fit curve (red) for each population was produced by finding the minimum residual sum of squares of a curve with the equation $f(x)=A*\exp[-(x-\mu)^2/2\sigma^2]$ where A is the maximum, μ is the mean, and σ is the standard deviation. The goodness of fit was estimated by an R^2 coefficient of determination between the observed and fit values. Note that in (C) the largest residuals from the fit were from proteins with a higher observed abundance in the *P. falciparum* sample versus the *P. vivax* dataset. More proteins and peptide spectrum matches were observed in the *P. falciparum* sample, suggesting that these differences were due to limit of detection.
