# Supplementary material for: Proteogenomic analysis of the total and surface-exposed proteomes of Plasmodium vivax salivary gland sporozoites
Source: PLoS Negl Trop Dis. 2017 Jul 31;11(7):e0005791. doi: 10.1371/journal.pntd.0005791 (PMC5552340; doi:10.1371/journal.pntd.0005791)
Supplement: S4 Fig — (PDF) [file pntd.0005791.s006.pdf]

A

***P. vivax* thrombospondin-related anonymous protein (TRAP)**

MKLLQNKSYLLVVFLLYVSIFARGDEKVVDEVKYSEEVCNESVDLYLLVDGSGSIGYPNWITKVIPMLNG
LINSLSLSRDTINLYMNLFGNYTTELIRLGSGQSIDKRQALSKVTELRKSYSPYGTTNMTAALDEVQKHL
NDRVNREKAIQLVILMTDGIPNSKYTALEVAKKLKQRNVSLAVIGIGQGINHQFNRLIAGCRPRETNCKF
YSYADWNEAVALIKPFIAKVCTEVERVANCGPWDPWTACSVTCGRGTHSRSRPSLHEGCTTHMVSECEEG
ECPVEPEPLPVPAPLPTVPEDVNPRDTDDENENPNFNKGLDVPDEDDDEVPPANERADGNPVEENVFPPA
DDSVPDESNVLPLPPAVPGGSSEEFPADVQNNPDSPEELPMEQEVPQDNNVNEPERSDSKGYGVNEKVIP
NPLDNERDMANKNKTVHPGRKDSARDRYARPHGSTHVNNNRANANSDIPNNPVPSDYEQPEDKAKKSSNN
GYKIAGGVIAGLALVGCVGFAYNFVAGGGAAGMAGEPAPFDEAMAEDEKDVAEADQFKLPEDNDWN

Peptide detected by mass spectrometry in either sample
P. vivax *P01 reference sequence differs from* P. vivax *Sal-1 reference sequence*
Sequence variant detected that is found in field isolates but neither P01 nor Sal-1 reference proteome

B

**VK247**	100%	SYSPYGTTNMTAALDEVQ
**VK210**	42%	SYSPYGTTNMTAALDEVQ
	58%	SYSPYGTTNMTAALEEVQ
	0.5%	SYSPYGTTNMTAALDEVR

**VK247**	100%	VANCGPWDPWTACSVTCGK
**VK210**	20%	VANCGPWDPWTACSVTCGK
	80%	VANCGPWDPWTACSVTCGR

**VK247**	100%	AIQLVILMTDGIPNSK
**VK210**	63%	AIQLVILMTDGIPNSK
	37%	AIQLVILMTDGVPNSK

**VK247**	100%	SDSNGYGVNEK
**VK210**	100%	SDSNGYGVNEK
	0%	SDSKGYGVNEK

**VK247**	100%	YTALEVAKK
**VK210**	65%	YTALEVAKK
	35%	YTALEVANK

**VK247**	100%	ANENSDIPNNPVPSDYEQPEDK
**VK210**	81%	ANENSDIPNNPVPSDYEQPEDK
	19%	ANANSDIPNNPVPSDYEQPEDK

VK247: Peptide detected in VK247 whole proteome sample
VK210: Peptide detected in VK210 whole proteome sample. Relative amount of each peptide variant is given as %.
Sequence is conserved between P. vivax *P01* and P. vivax *Sal-1.*
Sequence variant found in P. vivax *P01 but not* P. vivax *Sal-1*
Sequence variant found in P. vivax *Sal-1 but not in* P. vivax *P01*
Sequence variant only found in field isolates

S4 Fig. Mass spectrometry reveals sequence polymorphisms in thrombospondin-related anonymous protein (TRAP). (A) The sequence of TRAP from the *P. vivax* P01 reference proteome is shown. Residues in red text were detected by mass spectrometry in either the VK210 or the VK247 sample. Residues shaded in blue are amino acid substitutions found in the *P. vivax* P01 reference proteome that differ from the *P. vivax* Sal-1 reference proteome. Residues shaded in green are amino acids for which substitutions were observed in the samples that were not found in either reference proteome but were found in Thai field isolates by DNA-seq or RNA-seq. Observed sequence polymorphisms are detailed in S5 Table and in (B) the distribution of multiple sequence polymorphisms in TRAP identified in the whole proteome samples. As was observed with CSP (S3 Fig), it appeared that the VK247 sample contained a single variant of TRAP, whereas the VK210 sample contained a mixture of at least two TRAP variants. The percent composition of each polymorphism-containing peptide was estimated as the proportion of peptide spectrum matches identifying that peptide.
