# Supplementary material for: Proteogenomic analysis of the total and surface-exposed proteomes of Plasmodium vivax salivary gland sporozoites
Source: PLoS Negl Trop Dis. 2017 Jul 31;11(7):e0005791. doi: 10.1371/journal.pntd.0005791 (PMC5552340; doi:10.1371/journal.pntd.0005791)
Supplement: S5 Fig — (PDF) [file pntd.0005791.s007.pdf]

A

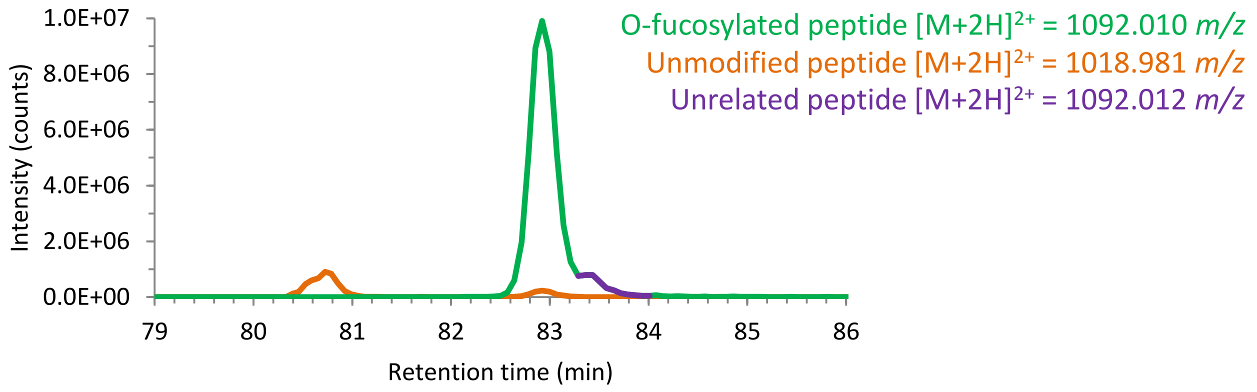

B

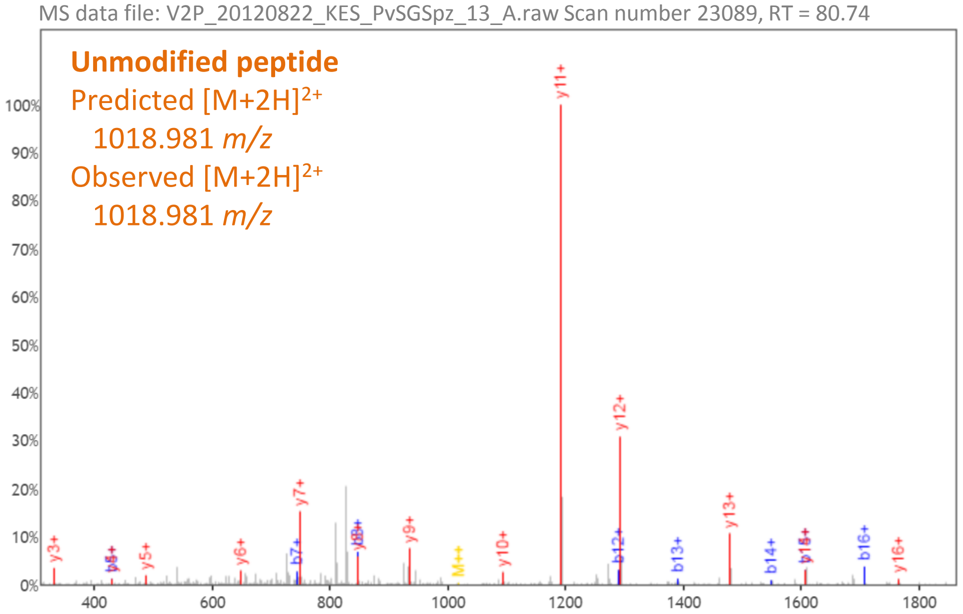

b+	#	Seq	#	y+
72.0444	1	A	19	
173.0921	2	T	18	1965.9157
272.1605	3	V	17	1864.8680
329.1819	4	G	16	1765.7996
430.2296	5	T	15	1708.7782
559.2722	6	E	14	1607.7305
745.3515	7	W	13	1478.6879
846.3992	8	T	12	1292.6086
943.4520	9	P	11	1191.5609
1103.4826	10	C	10	1094.5081
1190.5146	11	S	9	934.4775
1289.5831	12	V	8	847.4454
1390.6307	13	T	7	748.3770
1550.6614	14	C	6	647.3294
1607.6829	15	G	5	487.2987
1706.7513	16	V	4	430.2772
1763.7727	17	G	3	331.2088
1862.8411	18	V	2	274.1874
	19	R	1	175.1190

C

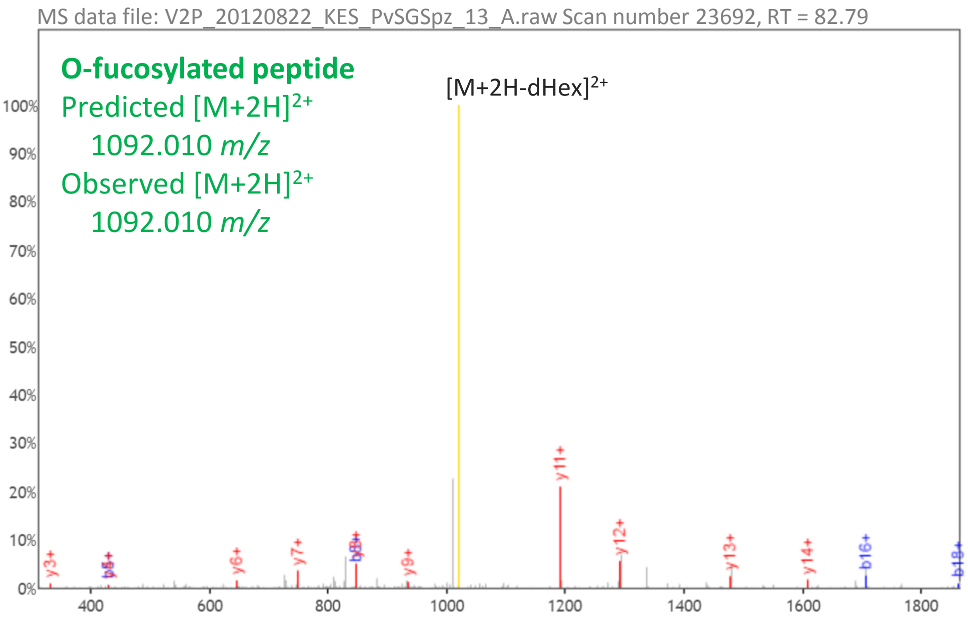

b+	#	Seq	#	y+
72.0444	1	A	19	
173.0921	2	T	18	1965.9157
272.1605	3	V	17	1864.8680
329.1819	4	G	16	1765.7996
430.2296	5	T	15	1708.7782
559.2722	6	E	14	1607.7305
745.3515	7	W	13	1478.6879
846.3992	8	T	12	1292.6086
943.4520	9	P	11	1191.5609
1103.4826	10	C	10	1094.5081
1190.5146	11	S	9	934.4775
1289.5831	12	V	8	847.4454
1390.6307	13	T	7	748.3770
1550.6614	14	C	6	647.3294
1607.6829	15	G	5	487.2987
1706.7513	16	V	4	430.2772
1763.7727	17	G	3	331.2088
1862.8411	18	V	2	274.1874
	19	R	1	175.1190

S5 Fig. Evidence for glycosylation of CSP in *P. vivax* VK210 salivary gland sporozoites. (A) Representative extracted ion chromatograms (XIC) of the doubly-charged ions of the CSP peptide ATVGTEWTPCSVTCGVGVR from *P. vivax* VK210 salivary gland sporozoites. The largest chromatographic peak (green) was produced by a species with a mass matching the peptide plus a deoxyhexose, which we presume to be O-fucose. An unidentified peptide of similar mass (purple) created a shoulder peak. Though the MS2 spectrum for this species was not confidently mapped to a peptide sequence, it was distinct from the identifying spectra of the CSP peptide (not shown; scan number 23860). The unmodified peptide (orange) was observed co-eluting with the putatively-fucosylated species, consistent with loss of the gas-phase-labile modification due to collision-induced dissociation (CID) within the mass analyzer source, as well as eluting as a distinct peak at 80.7 min. These data suggest that the majority of CSP in the sample was modified with a single O-fucose, but that some unmodified CSP was also present. Representative collision-induced dissociation (CID) fragmentation spectra of the unmodified (B) and glycosylated (C) peptides confirmed the peptide sequence. CID of the peptide lacking O-fucose (B) provided confirmation of the assignments of the fragment spectra obtained from the O-fucosylated species (C). Fragment ions are annotated as b-ions (blue) and y-ions (red). The unfragmented peptide is designated M (yellow) with addition of protons (H) and neutral loss of deoxyhexose (dHex). Fucose is a deoxyhexose. Fragmentation spectra of the O-fucosylated peptide showed that the dominant product of CID fragmentation was the intact peptide that had lost the O-linked glycan. No peptide fragment spectra were observed with the O-fucose glycan intact, so the location of the glycan could not be confirmed, but based on the presence of the O-fucosylation motif, we presume that the Thr of CSVTCG was modified.
