# Supplementary material for: Proteogenomic analysis of the total and surface-exposed proteomes of Plasmodium vivax salivary gland sporozoites
Source: PLoS Negl Trop Dis. 2017 Jul 31;11(7):e0005791. doi: 10.1371/journal.pntd.0005791 (PMC5552340; doi:10.1371/journal.pntd.0005791)
Supplement: S7 Fig — (PDF) [file pntd.0005791.s009.pdf]

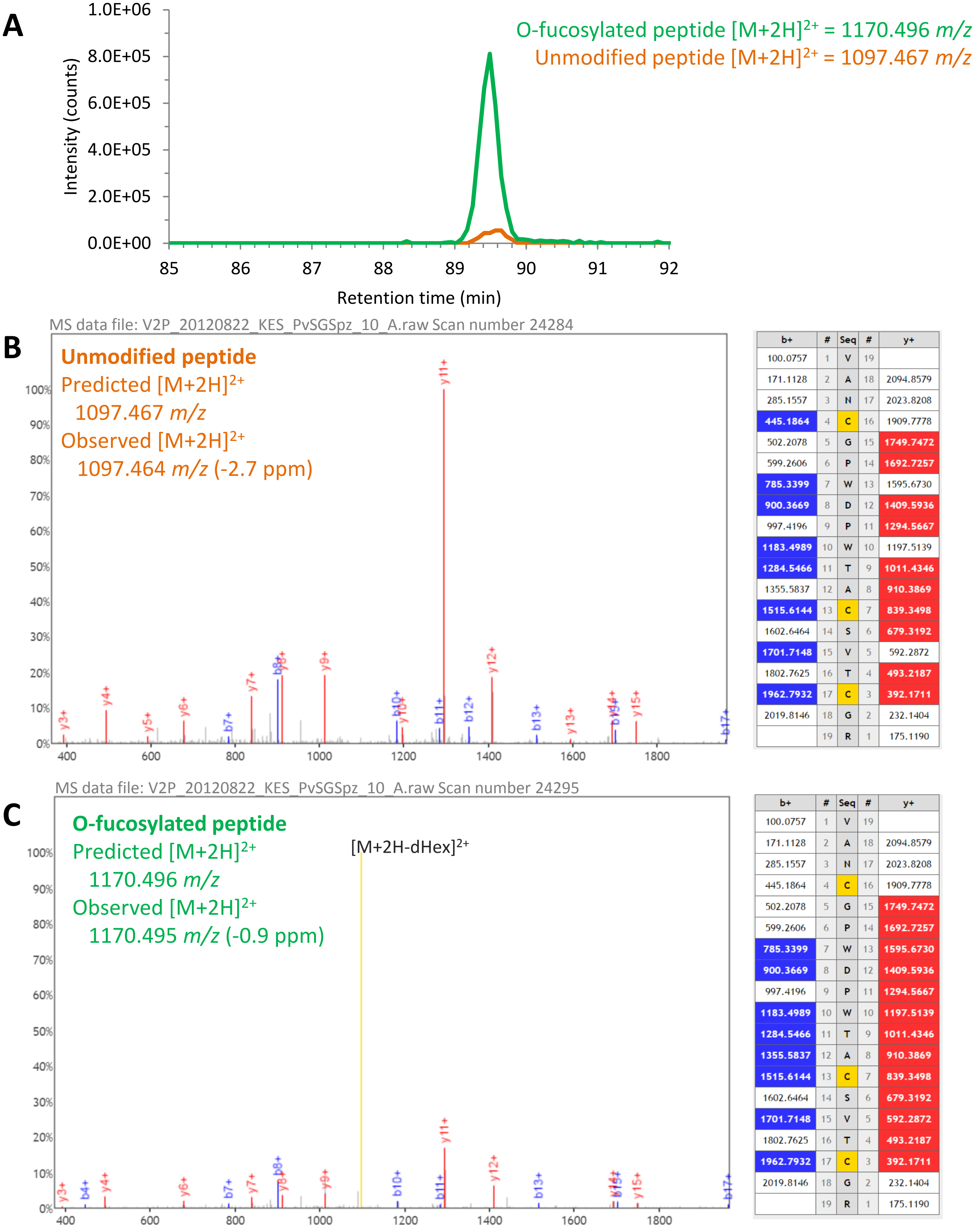

b+	#	Seq	#	y+
100.0757	1	V	19	
171.1128	2	A	18	2094.8579
285.1557	3	N	17	2023.8208
445.1864	4	C	16	1909.7778
502.2078	5	G	15	1749.7472
599.2606	6	P	14	1692.7257
785.3399	7	W	13	1595.6730
900.3669	8	D	12	1409.5936
997.4196	9	P	11	1294.5667
1183.4989	10	W	10	1197.5139
1284.5466	11	T	9	1011.4346
1355.5837	12	A	8	910.3869
1515.6144	13	C	7	839.3498
1602.6464	14	S	6	679.3192
1701.7148	15	V	5	592.2872
1802.7625	16	T	4	493.2187
1962.7932	17	C	3	392.1711
2019.8146	18	G	2	232.1404
	19	R	1	175.1190

b+	#	Seq	#	y+
100.0757	1	V	19	
171.1128	2	A	18	2094.8579
285.1557	3	N	17	2023.8208
445.1864	4	C	16	1909.7778
502.2078	5	G	15	1749.7472
599.2606	6	P	14	1692.7257
785.3399	7	W	13	1595.6730
900.3669	8	D	12	1409.5936
997.4196	9	P	11	1294.5667
1183.4989	10	W	10	1197.5139
1284.5466	11	T	9	1011.4346
1355.5837	12	A	8	910.3869
1515.6144	13	C	7	839.3498
1602.6464	14	S	6	679.3192
1701.7148	15	V	5	592.2872
1802.7625	16	T	4	493.2187
1962.7932	17	C	3	392.1711
2019.8146	18	G	2	232.1404
	19	R	1	175.1190

S7 Fig. Evidence for glycosylation of TRAP in *P. vivax* VK210 salivary gland sporozoites. (A) Representative extracted ion chromatograms (XIC) of the doubly-charged ions of the TRAP peptide VANCGPWDPWTACSVTCGR from *P. vivax* VK210 salivary gland sporozoites. The largest chromatographic peak (green) was produced by a species with a mass matching the peptide plus a deoxyhexose, which we presume to be O-fucose. The unmodified peptide was observed co-eluting with the putatively-fucosylated species, consistent with loss of the gas-phase-labile modification due to collision-induced dissociation (CID) within the mass analyzer source. Unlike for CSP (S5 Fig and S6 Fig), the unmodified peptide was not observed except arising from neutral loss of the glycan, suggesting that all or nearly all of the TRAP present in the sample was glycosylated. Representative collision-induced dissociation (CID) fragmentation spectra of the unmodified (B) and glycosylated (C) peptides confirmed the peptide sequence. CID of the peptide lacking O-fucose (B) provided confirmation of the assignments of the fragment spectra obtained from the O-fucosylated species (C). Fragment ions are annotated as b-ions (blue) and y-ions (red). The unfragmented peptide is designated M (yellow) with addition of protons (H) and neutral loss of deoxyhexose (dHex). Fucose is a deoxyhexose. Fragmentation spectra of the O-fucosylated peptide showed that the dominant product of CID fragmentation was the intact peptide that had lost the O-linked glycan. No peptide fragment spectra were observed with the O-fucose glycan intact, so the location of the glycan could not be confirmed, but based on the presence of the O-fucosylation motif, we presume that the Thr of CSVTCG was modified.
