# Supplementary material for: Proteogenomic analysis of the total and surface-exposed proteomes of Plasmodium vivax salivary gland sporozoites
Source: PLoS Negl Trop Dis. 2017 Jul 31;11(7):e0005791. doi: 10.1371/journal.pntd.0005791 (PMC5552340; doi:10.1371/journal.pntd.0005791)
Supplement: S8 Fig — (PDF) [file pntd.0005791.s010.pdf]

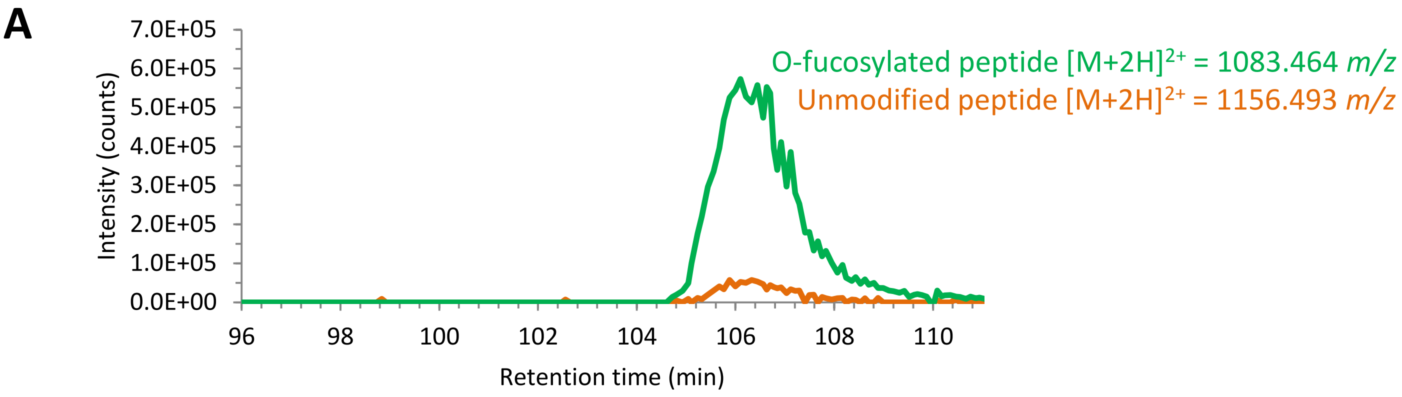

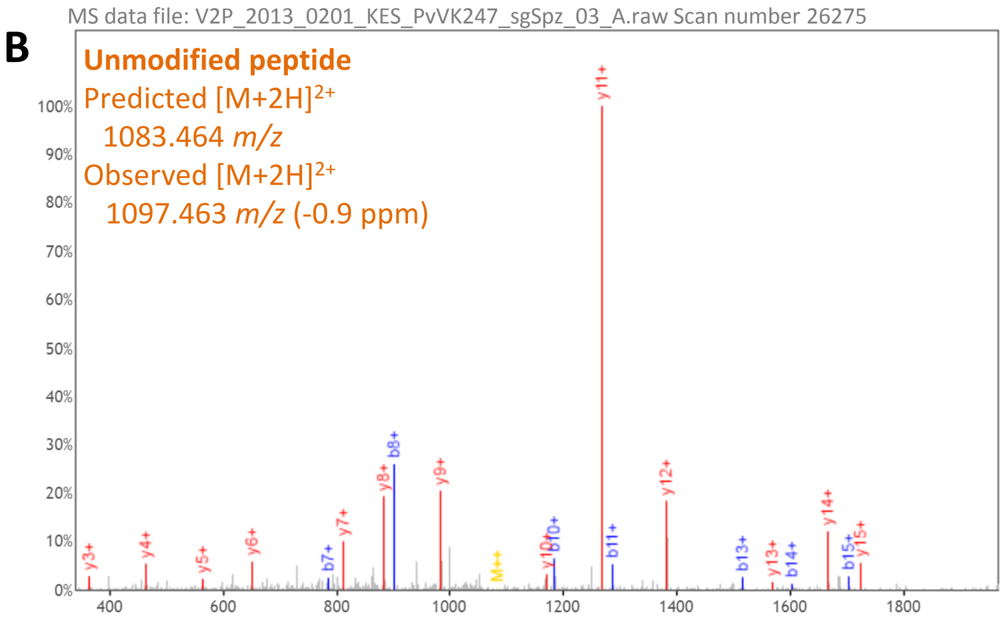

b+	#	Seq	#	y+
100.0757	1	V	19	
171.1128	2	A	18	2066.8517
285.1557	3	N	17	1995.8146
445.1864	4	C	16	1881.7717
502.2078	5	G	15	1721.7410
599.2606	6	P	14	1664.7196
785.3399	7	W	13	1567.6668
900.3669	8	D	12	1381.5875
997.4196	9	P	11	1266.5606
1183.4989	10	W	10	1169.5078
1284.5466	11	T	9	983.4285
1355.5837	12	A	8	882.3808
1515.6144	13	C	7	811.3437
1602.6464	14	S	6	651.3130
1701.7148	15	V	5	564.2810
1802.7625	16	T	4	465.2126
1962.7932	17	C	3	364.1649
2019.8146	18	G	2	204.1343
	19	K	1	147.1128

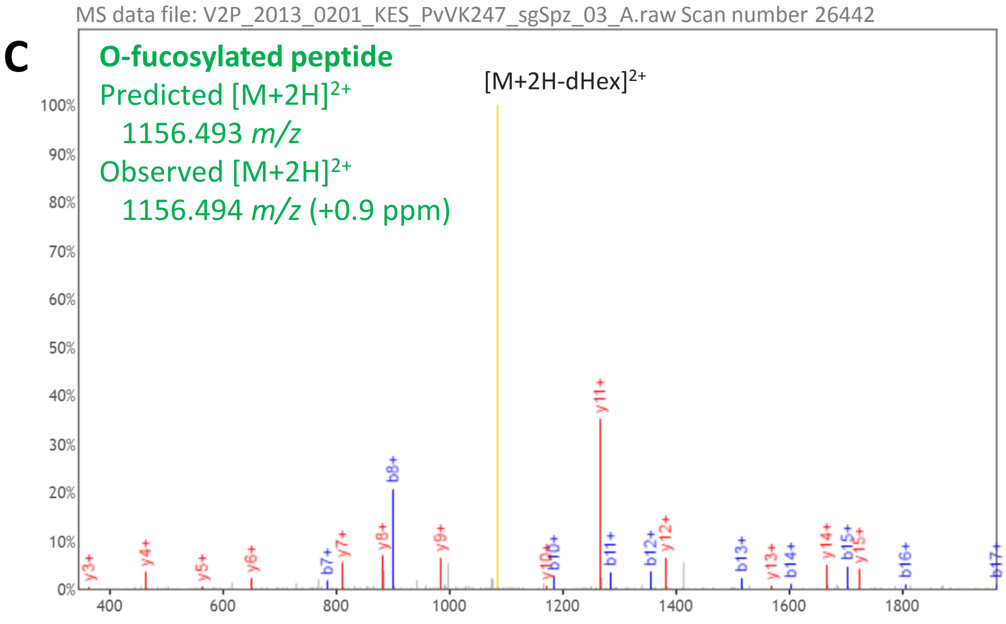

b+	#	Seq	#	y+
100.0757	1	V	19	
171.1128	2	A	18	2066.8517
285.1557	3	N	17	1995.8146
445.1864	4	C	16	1881.7717
502.2078	5	G	15	1721.7410
599.2606	6	P	14	1664.7196
785.3399	7	W	13	1567.6668
900.3669	8	D	12	1381.5875
997.4196	9	P	11	1266.5606
1183.4989	10	W	10	1169.5078
1284.5466	11	T	9	983.4285
1355.5837	12	A	8	882.3808
1515.6144	13	C	7	811.3437
1602.6464	14	S	6	651.3130
1701.7148	15	V	5	564.2810
1802.7625	16	T	4	465.2126
1962.7932	17	C	3	364.1649
2019.8146	18	G	2	204.1343
	19	K	1	147.1128

S8 Fig. Evidence for glycosylation of TRAP in *P. vivax* VK247 salivary gland sporozoites. (A) Representative extracted ion chromatograms (XIC) of the doubly-charged ions of the TRAP peptide VANCGPWDPWTACSVTCGK from *P. vivax* VK247 salivary gland sporozoites. The largest chromatographic peak (green) was produced by a species with a mass matching the peptide plus a deoxyhexose, which we presume to be O-fucose. The unmodified peptide was observed co-eluting with the putatively-fucosylated species, consistent with loss of the gas-phase-labile modification due to collision-induced dissociation (CID) within the mass analyzer source. Unlike for CSP (S5 Fig and S6 Fig), the unmodified peptide was not observed except arising from neutral loss of the glycan, suggesting that all or nearly all of the TRAP present in the sample was glycosylated. Representative collision-induced dissociation (CID) fragmentation spectra of the unmodified (B) and glycosylated (C) peptides confirmed the peptide sequence. CID of the peptide lacking O-fucose (B) provided confirmation of the assignments of the fragment spectra obtained from the O-fucosylated species (C). Fragment ions are annotated as b-ions (blue) and y-ions (red). The unfragmented peptide is designated M (yellow) with addition of protons (H) and neutral loss of deoxyhexose (dHex). Fucose is a deoxyhexose. Fragmentation spectra of the O-fucosylated peptide showed that the dominant product of CID fragmentation was the intact peptide that had lost the O-linked glycan. No peptide fragment spectra were observed with the O-fucose glycan intact, so the location of the glycan could not be confirmed, but based on the presence of the O-fucosylation motif, we presume that the Thr of CSVTCG was modified.
