# Supplementary material for: Proteogenomic analysis of the total and surface-exposed proteomes of Plasmodium vivax salivary gland sporozoites
Source: PLoS Negl Trop Dis. 2017 Jul 31;11(7):e0005791. doi: 10.1371/journal.pntd.0005791 (PMC5552340; doi:10.1371/journal.pntd.0005791)
Supplement: S3 Fig — (PDF) [file pntd.0005791.s005.pdf]

A. Circumsporozoite protein identified in the VK210 sample

Shared N-terminal sequence

MKNFILLAVSSILLVDLFPTHCGHNVDLSKAINLNGVNFNNVDASSLGAAHVGQSASRGRGL**GENPDDEEGDAKK**KKDGKKAEPKNPR
ENK

VK210 Repeat Region

LK**QPAGDRADGQPAGDRADGQPAGDRADGQPAGDRAAGQPAGDRADGQPAGDRAAGQPAGDRADGQPAGDRAAGQPAGDRADGQPAGD**
RAAGQPAGDRAAGQPAGDRADGQPAGDRAAGQPAGDRADGQPAGDRAAGQPAGDRAAGQPAGNGAGGQAAGGNAGGNAGGNAGGNAGG
QGQNNEGANAPNEKSVK

VK247 Repeat Region

LKQPEDGAGNQPGANGAGNQPGANGAGNQPGANGAGNQPGANGAGNQPGANGAGNQPGANGAGNQPGANGAGNQPGANGADDQPGANG
AGNQPGANGAGNQPGANGAGNQPGANGAGDQPGANGAGNQPGANGAGDQPGANGAGNQPGANGAGNQPGANEAGNQPGANGAGNQPGA
NGAGNQPGANGAGGQAAGGNAANKKAGD**AGAGQGQNNEGANAPNEK**SVK
*

Shared C-terminal sequence

EYLDKVR**ATVGTEWTPCSVTCGVGVR**VRRRVNAAN**KKPEDLTLNDLETDVCTMDK**CAGIFNVVSNSLGLVILLVLALFN

B. Circumsporozoite protein identified in the VK247 sample

Shared N-terminal sequence

MKNFILLAVSSILLVDLFPTHCGHNVDLSKAINLNGVNFNNVDASSLGAAHVGQSASRGRGL**GENPDDEEGDAKK**KKDGKKAEPKNPR
ENK

VK210 Repeat Region

LKQPAGDRADGQPAGDRADGQPAGDRADGQPAGDRAAGQPAGDRADGQPAGDRAAGQPAGDRADGQPAGDRAAGQPAGDRADGQPAGD
RAAGQPAGDRAAGQPAGDRADGQPAGDRAAGQPAGDRADGQPAGDRAAGQPAGDRAAGQPAGNGAGGQAAGGNAGGNAGGNAGGN**AGG**
QGQNNEGANAPNEKSVK
#

VK247 Repeat Region

LKQPEDGAGNQPGANGAGNQPGANGAGNQPGANGAGNQPGANGAGNQPGANGAGNQPGANGAGNQPGANGAGNQPGANGADDQPGANG
AGNQPGANGAGNQPGANGAGNQPGANGAGDQPGANGAGNQPGANGAGDQPGANGAGNQPGANGAGNQPGANEAGNQPGANGAGNQPGA
N**GAGNQPGANGAGGQAAGGNAANKKAGDAGAGQGQNNEGANAPNEKSVK**

Shared C-terminal sequence

EYLDKVRATVGTEWTPCSVTCGVGVRVRR**RVNAANKKPEDLTLNDLETDVCTMDK**CAGIFNVVSNSLGLVILLVLALFN

S3 Fig. Mass spectral evidence for circumsporozoite protein haplotype. The sequence of circumsporozoite protein is shown as found in the *P. vivax* P01 reference proteome (PVP01_0835600). The C-terminal and N-terminal sequences are conserved between the VK210 and VK247 haplotypes, but the tandem repeat region is distinct between the two. Red text indicates that the sequence was positively identified by mass spectrometry from peptide spectrum matches (PSM) with PeptideProphet probabilities corresponding to a false discovery rate less than one percent from (A) the VK210 whole proteome sample and (B) the VK247 whole proteome sample. In (A) the VK210 sample, 75 PSM identified semi-tryptic peptides matching the VK247-specific sequence at the C-terminal end of the tandem repeat section (*), while 192 PSM identified the VK210 version of this sequence. It is notable that in the VK247 sample in (B), the fully-tryptic peptide at this location was the dominant species, which was not observed in the VK210 sample. Interestingly, 19 of the 192 PSM identifying the VK210-specific version of this sequence (10%) identified the peptide **A**AGGNAGGQGQNNEGANAPNEK, which is found in *P. vivax* Sal-1 CSP, while 112 PSM (58%) contained the *P. vivax* P01 version **N**AGGNAGGQGQNNEGANAPNEK. The remaining PSM identifying VK210-specific peptides from this region did not contain the discriminating residue. An additional two PSM were observed for the peptide AGG**D**AGGQGQNNEGANAPNEK, a variant observed in field isolates that contains a Asn→Asp substitution in the VK210-specific peptide AGG**N**AGGQGQNNEGANAPNEK. This polymorphism could also be explained by deamidation of the peptide during sample handling. Given high degree of polymorphism in the CSP repeat region, it is entirely possible that these peptide sequences could be explained by a different CSP sequence not accounted for here. At the least, these observations are consistent with a mixed infection of more than one strain of *P. vivax*. Similar evidence was seen for TRAP (S4 Fig). For the VK247 sample (B), fewer haplotype-specific peptides were identified since the repeat region lacked Lys and Arg residues necessary to produce tryptic peptides. However, a peptide specific to the VK247 sequence in the repeat region was identified from 774 PSM, including 607 PSM from fully tryptic peptides (as opposed to the semi-tryptic peptide fragments identifying the putative mixed infection in the VK210 sample).Three PSM mapped to the semi-tryptic VK210 peptide **AG**GQGQNNEGANAPNEKSVK (marked with a #). However, this peptide is isobaric with the VK247 peptide **GA**GQGQNNEGANAPNEKSVK, and the identifying MS2 lacked the identifying b1, b2, y19, and y20 ions to distinguish between them. Combined with the total absence of other VK210-specific peptides, which are otherwise easily detectable as evidenced in (A), it appears that this sample contained pure VK247 CSP. Based on this and the evidence for TRAP (S2 Fig), the VK247 sample appears to have contained a single field isolate and not a mixed infection.
